# Syntheses and Electrochemical and EPR Studies of Porphyrins Functionalized with Bulky Aromatic Amine Donors

**DOI:** 10.3390/molecules28114405

**Published:** 2023-05-29

**Authors:** Mary-Ambre Carvalho, Khalissa Merahi, Julien Haumesser, Ana Mafalda Vaz Martins Pereira, Nathalie Parizel, Jean Weiss, Maylis Orio, Vincent Maurel, Laurent Ruhlmann, Sylvie Choua, Romain Ruppert

**Affiliations:** 1 Institut de Chimie, UMR CNRS 7177, Université de Strasbourg, Institut Le Bel, 4 rue Blaise Pascal, 67000 Strasbourg, France; 2 Campus of St Jérôme, Aix-Marseille University, CNRS, Centrale Marseille, iSm2, CEDEX 20, 13397 Marseille, France; 3 SyMMES, UMR 5819 CEA Grenoble/CNRS/Université Grenoble-Alpes, CEA Grenoble, 17 rue des Martyrs, CEDEX 9, 38054 Grenoble, France

**Keywords:** porphyrinoids, phenothiazine, radical cation, EPR spectroscopy, ENDOR, HYSCORE, spectroelectrochemistry

## Abstract

A series of nickel(II) porphyrins bearing one or two bulky nitrogen donors at the *meso* positions were prepared by using Ullmann methodology or more classical Buchwald–Hartwig amination reactions to create the new C-N bonds. For several new compounds, single crystals were obtained, and the X-ray structures were solved. The electrochemical data of these compounds are reported. For a few representative examples, spectroelectrochemical measurements were used to clarify the electron exchange process. In addition, a detailed electron paramagnetic resonance (EPR) study was performed to estimate the extent of delocalization of the generated radical cations. In particular, electron nuclear double resonance spectroscopy (ENDOR) was used to determine the coupling constants. DFT calculations were conducted to corroborate the EPR spectroscopic data.

## 1. Introduction

**Figure 1:**
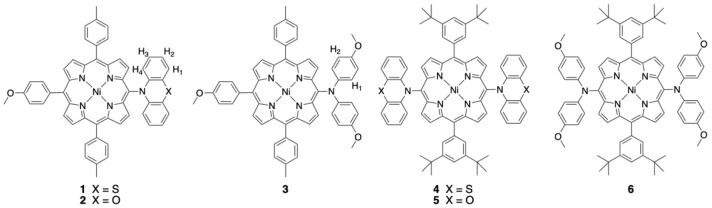
Chemical structures of the nickel(II) porphyrins studied.

The selective peripheral functionalization of the porphyrin macrocycle has a long history [1,2]. The introduction of some substituents remained a synthetic challenge until recently. More specifically, introducing nitrogen nucleophiles at the periphery of porphyrin was mainly developed in the last twenty years. Several approaches were proposed, with or without metal-catalyzed reactions [3,4,5,6,7], and, for example, the classical palladium-catalyzed Buchwald–Hartwig amination reaction worked well for nucleophilic amines [8,9,10,11]. Although the introduction of many functional groups was considered a solved problem, adding bulky and/or less-nucleophilic aromatic amines with good yields at the *meso* positions of porphyrins remained a synthetic challenge, and this problem was really tackled over the last decade. The so-called Pd-PEPPSI complexes were chosen by the group of Osuka to prepare porphyrins bearing aromatic amines at their *meso* positions, which could later be fused to the aromatic core of the porphyrin [12,13,14,15]. Additionally, inexpensive copper-catalyzed Ullmann couplings were used to make these C-N connections [16]. In this particular case, porphyrins could be substituted once or twice with phenoxazine or carbazole units in good yields [17]. Such reactions are particularly useful to introduce electron donors and/or acceptors to build new molecular dyads and triads for dye-sensitized solar cells (DSSCs) or to prepare model compounds mimicking elementary steps in natural photosynthetic systems [18,19,20,21,22]. Mixed-valence compounds, with porphyrins as large π-bridges and aromatic amines as redox centers, were also studied [23,24]. The compounds described in this manuscript are shown in Figure 1 and were studied by electrochemistry, EPR spectroscopy, and DFT calculations to obtain insight into the electronic delocalization of the generated radical cation of the amines on the π-systems of the nickel(II) porphyrins.

## 2. Results and Discussion

### 2.1. Syntheses and Characterization

**Scheme 1:**
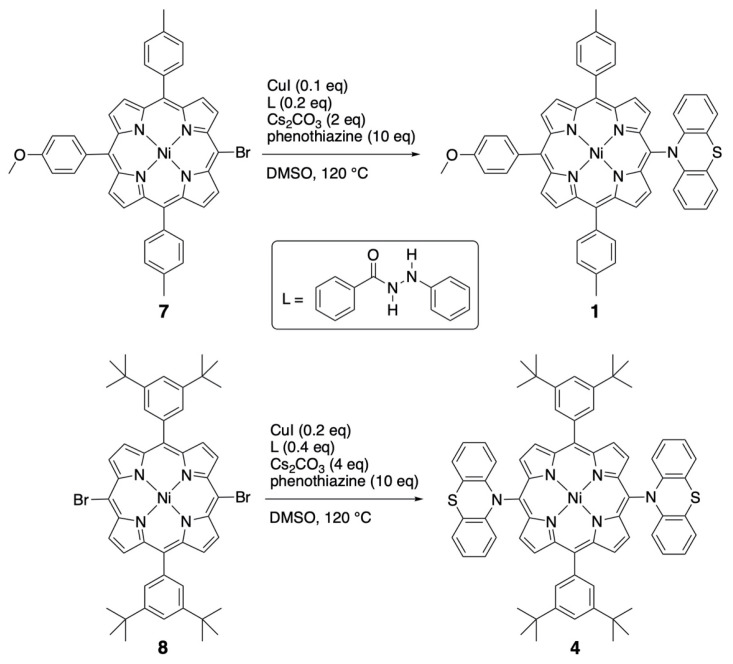
Preparation of the nickel(II) porphyrins bearing one or two *meso*-phenothiazine substituents.

Some compounds described in this study were already described earlier, but their preparation is shortly included in the synthetic procedures presented. The phenoxazine and phenothiazine-substituted porphyrins were obtained by the inexpensive Ullmann coupling between brominated porphyrin precursors and these amines (see Scheme 1) [17]. The dianisylamines were introduced by using a classical palladium-catalyzed Buchwald–Hartwig amination reaction [5].

**Figure 2:**
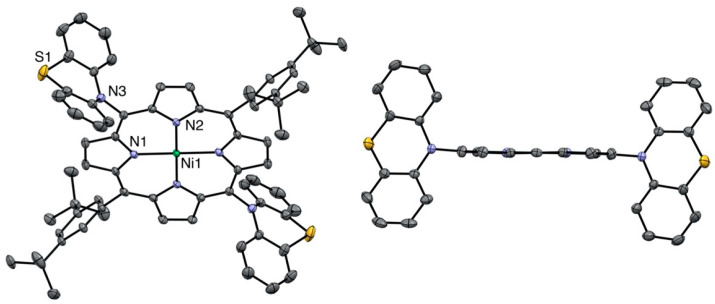
Two views of the nickel(II) porphyrin **4** X-ray structure (all hydrogen atoms are omitted for clarity). **Right**: View showing the planarity of the porphyrin plane (two *meso*-aryl groups omitted for clarity).

All compounds were characterized by standard spectroscopic techniques. Single crystals were obtained for some of them, and the structures were solved. The X-ray structure of the bis-phenothiazine-substituted nickel(II) porphyrin is presented in Figure 2. As noticed before in the solid-state structures of the 5,15-bis-carbazole- or 5,15-bis-phenoxazine-substituted nickel(II) porphyrins, the porphyrin plane is almost perfectly planar, despite the fact that the metal ion inside the aromatic cavity is nickel(II). Indeed, the vast majority of the nickel(II) porphyrin structures reported in the literature are strongly ruffled because the nickel(II) ion is too small for the inner porphyrin cavity [25].

**Figure 3:**
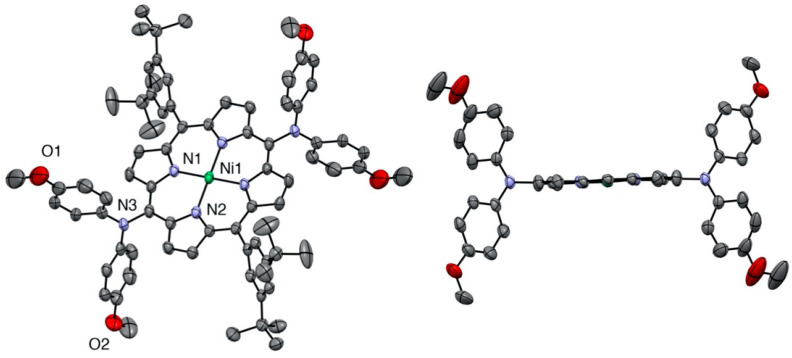
Two views of the nickel(II) porphyrin **6** X-ray structure (all hydrogen atoms are omitted for clarity). **Right**: View showing the planarity of the porphyrin plane (two *meso*-aryl groups omitted for clarity).

The same planarity of the porphyrin macrocycle was observed for the 5,15-bis(dianisylamine)nickel(II) porphyrin (see Figure 3).

**Figure 4:**
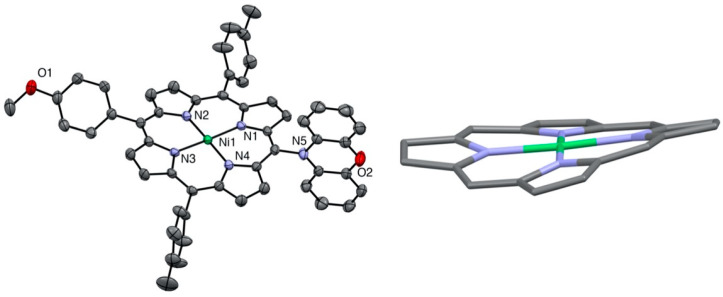
X-ray structure of nickel(II) porphyrin **2** (all hydrogen atoms are omitted for clarity). **Right**: view showing the ruffled nickel(II) porphyrin core.

The related nickel(II) porphyrin bearing only one phenoxazine donor was described earlier, but the X-ray structure was missing. In this case (see Figure 4), the macrocycle was slightly ruffled, and the Ni-N distances were shorter than in the two other examples (1.94–1.95 Å instead of 1.95–1.97 Å).

As expected for very bulky donors (phenoxazine or phenothiazine), the aromatic amines are not in the plane of the porphyrin and are almost orthogonal to this plane. The *meso*-carbon to nitrogen distances were all in the same range (1.43 to 1.44 Å). The main difference between dianisylamine and the two other donors resides in the flexibility of the two aromatic groups attached to the nitrogen, whereas in the case of phenoxazine and phenothiazine, the linking oxygen or sulfur atoms prevent rotation of the two phenyl groups.

### 2.2. Electrochemical Studies

Compounds **1**, **2**, **3**, **4**, **5**, and **6** were studied by cyclic voltammetry, and the results are summarized in Table 1.

**Figure 5:**
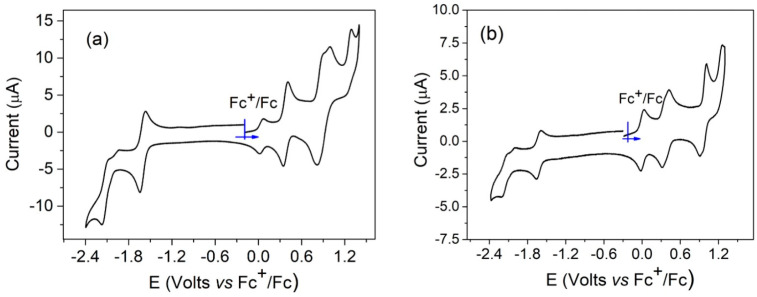
Cyclic voltammetry of compounds **1** (**a**) and **4** (**b**), recorded in dichloromethane, NBu_4_PF_6_ (0.1 M), scan rate = 0.1 V/s.

The reduction and oxidation steps of these molecules can be localized on the porphyrinic core and/or on the aromatic amine *meso*-substituents. In the case of nickel(II) porphyrins, the redox processes are localized on the aromatic moiety, and the nickel(II) ion remains formally in oxidation state 2. Generally, for nickel(II) porphyrins, two reduction and two oxidation states are present, and sometimes a third oxidation step can be observed [26]. For compounds **1**–**6**, one or two additional oxidation states were expected due to the presence of the aromatic amines. The electrochemical data for compounds **2** and **5** were described earlier [17]. The cyclic voltammetry of compounds **1** and **4** is shown in Figure 5. For both compounds, two reduction waves corresponding to the formation of the porphyrinic radical anion and dianion were observed. In oxidation, four and five oxidation steps were, respectively, observed for compounds **1** and **4**.

**Figure 6:**
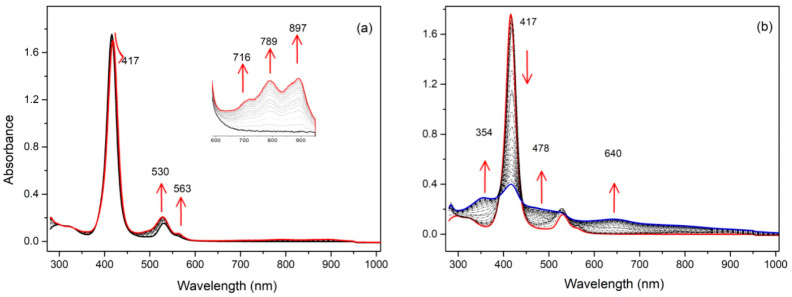
UV/Vis spectra during the stepwise electrochemical oxidation of **1**: (**a**) at the first oxidation potential; (**b**) at the second oxidation potential (recorded in dichloromethane, 0.1 M NBu_4_PF_6_).

To ascertain the electronic states of the oxidized species of the compounds, spectroelectrochemical studies were carried out. For the monosubstituted porphyrin **1**, the first oxidation led to a new compound with almost no changes in the Soret band (from 415 to 417 nm) or the Q bands (see Figure 6). Three new bands appeared at 716, 789, and 897 nm. The very small bathochromic shift of the Soret band clearly indicated that the porphyrin ring was not involved in this redox process, and the new bands were attributed to a radical cation located on the substituent. Under the same conditions, increasing the oxidation potential to a value higher than the second oxidation modified the spectrum drastically. The intensity of the Soret band decreased drastically, showing that now the formation of the porphyrinic π-cation radical was occurring. The same behavior was observed for compound **2** (see Figure S10).

**Figure 7:**
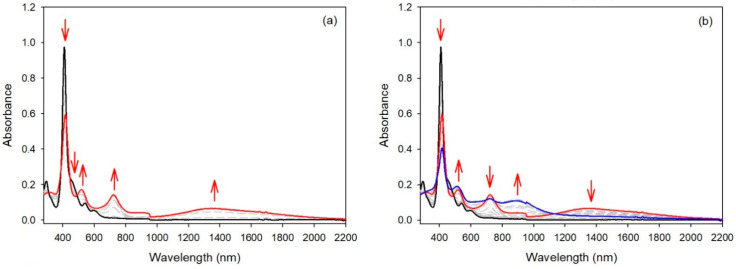
UV/Vis spectra during the stepwise electrochemical oxidation of **3**: (**a**) at the first oxidation potential; (**b**) at the second oxidation potential (recorded in dichloromethane, 0.1 M NBu_4_PF_6_).

Spectroelectrochemical studies of compound **3** under similar conditions led to a different result. While for compounds **1** and **2** the intensities of the Soret bands remained quasi-identical after the first oxidation step, the intensity of the Soret band of compound **3** dropped markedly after the first oxidation step (see Figure 7). In this case, the generation of the radical cation of the *meso*-donor nitrogen atom was partly delocalized over the aromatic porphyrinic moiety. For the three compounds bearing only one *meso*-donor group, the second oxidation step led to a large decrease in the Soret intensity, showing that the second oxidation steps were localized on the porphyrin aromatic ring.

**Figure 8:**
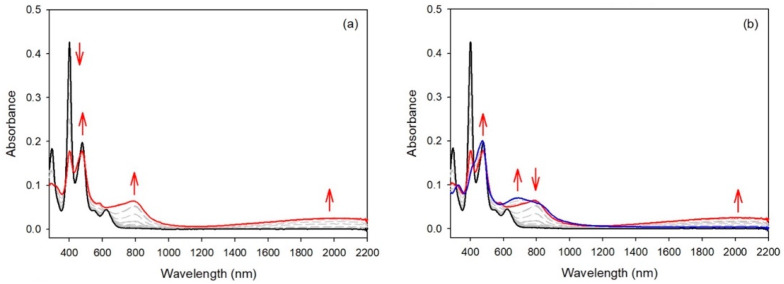
UV/Vis spectra during the stepwise electrochemical oxidation of **6**: (**a**) at the first oxidation potential; (**b**) at the second oxidation potential (recorded in dichloromethane, 0.1 M NBu_4_PF_6_).

The cyclic voltammetry of compound **4** (see Figure 5 right) was similar to the results described earlier for compound **5** and a bis-carbazole-substituted nickel(II) porphyrin, with small differences in potential values [17]. Again, compound **6**, bearing two *meso*-bis-anisylamine donor groups, showed a different electrochemical behavior. The first oxidation step was reversible, but the following oxidation steps proved to be irreversible. The spectroelectrochemical study showed again that the aromatic porphyrinic core was involved in the oxidation processes because the Soret band intensity dropped markedly (see Figure 8). To confirm these observations, an EPR study and DFT calculations were carried out (vide infra).

### 2.3. EPR Studies

#### 2.3.1. CW-EPR/ENDOR Measurements

**Figure 9:**
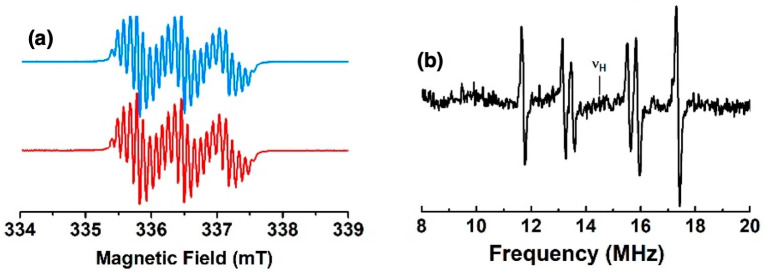
(**a**) X-band EPR spectrum of the radical cation of **1** in CH_2_Cl_2_ fluid solution at room temperature (blue) and its simulation (red); (**b**) ^1^H ENDOR spectrum in CH_2_Cl_2_ fluid solution at 200 K.

The X-band EPR spectra of the fluid solutions of the radicals from mono-substituted nickel(II) porphyrins generated by chemical oxidation with one equivalent of AgSbF_6_ in CH_2_Cl_2_ at room temperature are shown in Figure 1 and in Figure S1 in SI. They exhibit a well-resolved three-line pattern centered at g = 2.003 with nitrogen hyperfine coupling constants A_iso_(^14^N) of 19, 23, and 19 MHz for **1**, **2**, and **3**, respectively. Additionally, each of the three lines is resolved for **1** and **2** due to further hyperfine coupling interactions with magnetically active nuclei. To gain better information about the small spin density distributions within all compounds, we used CW electron nuclear double resonance spectroscopy (ENDOR) as an alternative tool for studying radicals with many overlapping and/or incompletely resolved EPR lines. Three, four, and two additional hyperfine coupling interactions centered around the nuclear frequency of the proton are observed for **1**, **2**, and **3,** respectively (see Figure 9, Figures S11 and S12, and Table 2). These patterns arise from the contributions of the known additional couplings described for the corresponding radical cations of phenothiazine and phenoxazine [27,28]. From these data, the EPR spectra were best simulated with the hyperfine coupling constants reported in Table 2, which were in close agreement with the DFT-computed values. Oxidations were also performed in situ electrochemically in the EPR cavity. The EPR spectra observed after a few minutes of electrolysis were identical to the spectra obtained after chemical oxidations.

#### 2.3.2. HYSCORE Results

**Figure 10:**
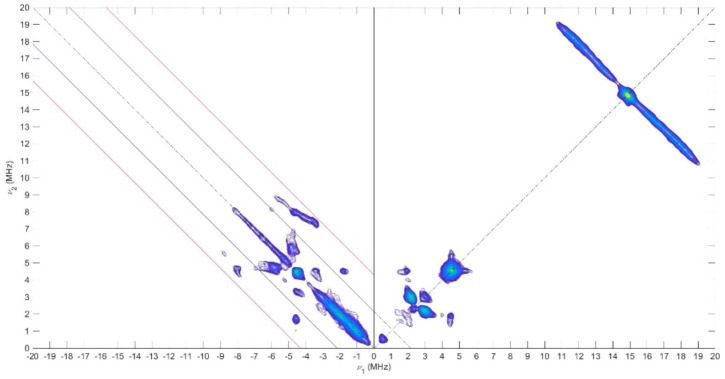
X-Band HYSCORE spectrum of **1** recorded at 70 K and collected at 349.4 mT, microwave frequency 9.70 GHz, τ = 136 ns.

Pulse EPR experiments were conducted using the 2D-Hyperfine sublevel correlation experiment (HYSCORE) to obtain information about the electronic structure and the delocalization of the unpaired electron all over the compounds. The use of HYSCORE spectroscopy affords the accurate assignment of various couplings with a large number of nuclei. For **1** and **2,** the HYSCORE spectra revealed in the (+, +) quadrant two distinct ridges centered at 14.5 MHz (the Larmor nuclear frequency of the ^1^H) with no symmetric cross-peak in the (−, +) quadrant (Figure 10 and Figure S13). These results demonstrated the presence of weak hyperfine constants (|A_iso_| < 2|ν_I_|) in agreement with the CW ^1^H-ENDOR experiments. At low frequency, the (+,+) quadrants showed nitrogen features (ν_I_ = 1.06 MHz), which correspond also to a weak coupling situation (|A_iso_| < 2|ν_I_|) with two pairs of cross peaks at 2.1 and 3.0 MHz for **1**, and are attributed to the porphyrin nitrogen atoms. In the (−, +) quadrant, a more complex pattern (i.e., the strong coupling situation where (|A_iso_| > 2|ν_I_|) is present and indicative of a hyperfine coupling with a more important anisotropy probably from a nitrogen nucleus. To interpret the low frequency part of the HYSCORE spectra, the ^14^N hyperfine and quadrupole data were computed, and these values were used as a starting point for the simulation. The corresponding ^14^N hyperfine and nuclear quadrupole couplings are given in Table 3 with the simulations shown in Figure S14 and support the presence of a second nitrogen atom with a hyperfine tensor and a quadrupole contribution very close to nitrogen from porphyrin with the phenothiazine and phenoxazine substituents. In contrast, the HYSCORE spectrum of **3** recorded at 80 K is rather different (Figure S13). A similar shape is observed at low frequency in the (+, +) quadrant, but the pattern visible in the (−, +) quadrant is different. This signal could originate from nitrogen atoms of the porphyrin with close hyperfine coupling values, as suggested by the DFT calculation. From this information, the following trends in the spin density distributions have emerged. The spin distribution over the Ni porphyrin is slightly different when species **1** and **2** are compared to **3**. For the latter, the computed values (see DFT calculations in SI) predict a non-zero spin density on the four ^14^N porphyrin, whereas only the two nitrogen atoms close to the *meso* group bear spin density for **1** and **2**. These results reflect the enhanced electron donating ability of the bis(*p*-anisyl)-amine substituent and the flexibility of this amine compared to the rigid phenoxazine and phenothiazine. Consequently, enhanced radical delocalization was observed in compound **3** when compared to compounds **1** and **2**.

#### 2.3.3. Magnetic Properties

**Figure 11:**
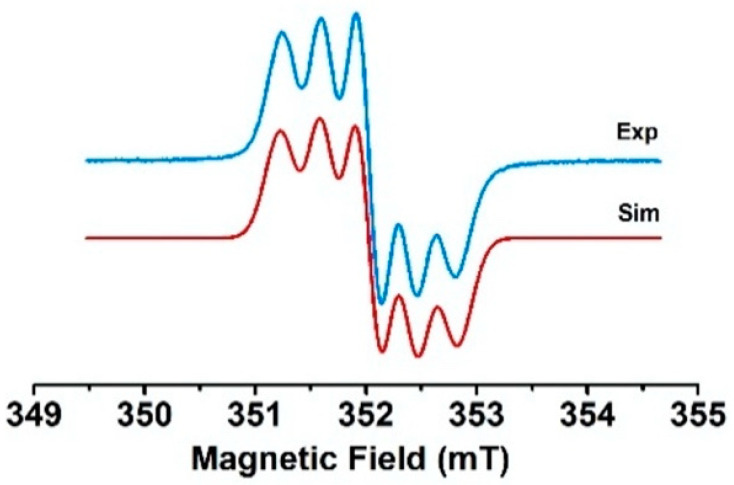
Experimental EPR spectrum of the dication of compound **4** in CH_2_Cl_2_ fluid solution at room temperature and its simulation.

The reaction of **4** and **5** with two equivalents of AgSbF_6_ in CH_2_Cl_2_ fluid solution yielded EPR spectra with five transitions consistent with two spin species and a ^14^N hyperfine coupling constant approximatively equal to the half of the hyperfine coupling constant of the monoradical species (see Figure 11 and Figure S16). This situation corresponds to the strong exchange limit and means that the exchange coupling is much higher than the hyperfine coupling constant. In contrast, one line was observed for **6** with a linewidth of 10 G and thus also suggested the formation of the biradical. The EPR spectra of the biradicals could also be obtained upon electrochemical generation if the electrolyses were carried out around the second oxidation potential. For compound **4**, a spectrum corresponding to a mixture of the mono- and biradical species (see Figure S17) was obtained. The very small differences between the first and second oxidation potentials for compounds **5** and **6** led directly to the doubly oxidized species, with EPR spectra identical to those obtained by chemical oxidation.

**Figure 12:**
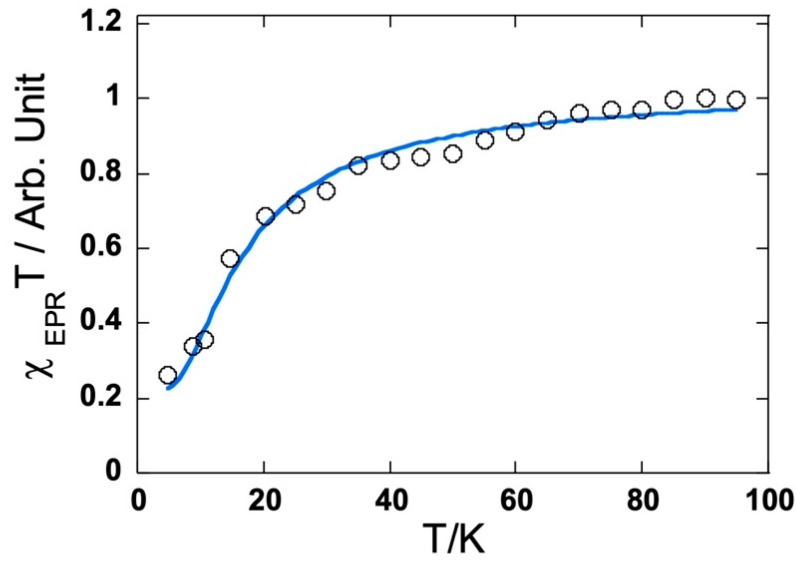
Temperature dependence of the EPR susceptibility (χT product) in CH_2_Cl_2_ frozen solution for the dication of **6**.

The frozen solution EPR spectra of three bis compounds recorded at 4 K exhibit only one intense symmetrical line (ΔB_PP_ = 12 G) centered on g = 2.00 without any fine structure characteristic of the triplet state, but a forbidden ΔMs = ±2 transition was detected at half field. This definitely indicates a biradical nature. To compare the exchange interaction, the temperature dependence of the EPR signal intensity (I) was measured. The intensity is known to be proportional to the magnetic susceptibility. Note that the EPR susceptibility is assessed as usual with the integrated intensity for the ΔMs = ±1 line with a high S/N ratio. This is correct as long as the line shape does not vary. In all three cases, the product IT decreases when the temperature is below 20 K, indicating the presence of an antiferromagnetic coupling (see Figure 12 for **6** and Figure S18). To obtain the exchange coupling constant (J/k_B_ where k_B_ is the Boltzmann constant), the observed curves were adjusted with a Bleaney–Bowers law with the additional contribution of a Curie law to account for the presence of the single radical. The estimated J/k_B_ values of −8 K for **4** and −8 K for **5** were very close, while the value of **6** was in the order of −30 K and showed much better magnetic communication between the two bis(*p*-anisyl)amine *meso*-substituents.

## 3. Materials and Methods

### 3.1. Materials

All commercial reagents (Aldrich, St. Louis, MI, USA; Fisher Scientific, Hampton, NH, USA; Merck, Rahway, NJ, USA) were used as received. All solvents were dried and freshly distilled before use. Column chromatography was performed using silica gel (Merck 0.40–0.63 nm).

### 3.2. Preparation of the Compounds

5,15-Diarylporphyrin and 5,10,15-triarylporphyrin were prepared by well-established porphyrin synthetic procedures, followed by bromination of the remaining free *meso* positions to obtain **7** and **8**. The syntheses and characterizations of these starting materials were described previously. Similarly, the syntheses of the porphyrins **2** and **5** bearing one or two phenoxazine donors at the *meso* positions were described previously [17].

**MonoPhenothiazine 1**. A degassed solution of brominated nickel(II) porphyrin **7** (53 mg, 72 μmol), phenothiazine (136 mg, 10 eq.), CuI (2 mg, 0.1 eq.), N′-phenylbenzoylhydrazine (3 mg, 0.2 eq.), Cs_2_CO_3_ (45 mg, 2 eq.) in DMSO (1 mL) was heated under argon at 120 °C for 16 h. After cooling, the reaction mixture was diluted in dichloromethane (100 mL), washed twice with a saturated NaHCO_3_ aqueous solution (50 mL), and finally twice with water. The organic phase was dried over Na_2_SO_4_, and the solvents evaporated. After chromatographic purification (silica gel, CH_2_Cl_2_ then CHCl_3_) and crystallization (CH_2_Cl_2_, n-hexane, MeOH), the desired compound **1** was isolated as a purple solid (18 mg, 21 μmol, 29%). ^1^H NMR (300 MHz, CDCl_3_, 25 °C), δ (ppm) = 9.29 (d, *J =* 5.0 Hz, 2H, pyrr.), 8.77 (d, *J =* 5.0 Hz, 2H, pyrr.), 8.74 (m, 4H, pyrr.), 7.91 (d, *J =* 8.6 Hz, 2H, H_o-Anis_), 7.87 (d, *J =* 7.9 Hz, 4H, H_o-tolyl_),7.45 (d, *J =* 7.9 Hz, 4H, H_m-tolyl_), 7.20 (d, *J =* 8.6 Hz, 2H, H_m-Anis_), 7.12 (dd, *J =* 7.6 and 1.5 Hz, 2H, H_a_), 6.73 (ddd, *J =* 7.6, 7.6, and 1.0 Hz, 2H, H_b_), 6.46 (ddd, *J =* 8.2, 7.6, and 1.5 Hz, 2H, H_c_), 5.79 (dd, *J =* 8.2 and 1.0 Hz, 2H, H_d_), 4.03 (s, 3H, OCH_3_), 2.62 (s, 6H, CH_3_). MS (Maldi-TOF, dithranol): Calcd for [C_53_H_37_N_5_NiOS]^+•^: 849.21; found 849.19. UV-Vis. (CH_2_Cl_2_): λ_max_ = 416 nm (ε = 24,5000 M^−1^.cm^−1^), 530 (19,000). Anal. Calcd for C_53_H_37_N_5_NiOS•1/2 CH_3_OH: C 74.14, H 4.54, N 8.08; found C 74.16, H 4.51, N 8.00.

**BisPhenothiazine 4**. A degassed solution of dibrominated nickel(II)porphyrin **8** (101 mg, 112 μmol), phenothiazine (220 mg, 10 eq.), CuI (4 mg, 0.2 eq.), N′-phenylbenzoylhydrazine (9 mg, 0.4 eq.), and Cs_2_CO_3_ (144 mg, 4 eq.) in DMSO (4 mL) was heated under argon at 120 °C for 16 h. After cooling, the reaction mixture was diluted in dichloromethane (100 mL), washed twice with a saturated NaHCO_3_ aqueous solution (50 mL), and finally twice with water. The organic phase was dried over Na_2_SO_4_, and the solvents evaporated. After chromatographic purification (silica gel, CH_2_Cl_2_ then CHCl_3_) and crystallization (CH_2_Cl_2_, n-hexane, MeOH), the desired compound **4** was isolated as a purple solid (32 mg, 28 μmol, 25%). ^1^H NMR (400 MHz, CDCl_3_, 25 °C), δ (ppm) = 9.38 (d, *J =* 5.0 Hz, 4H, pyrr.), 8.81 (d, *J =* 5.0 Hz, 4H, pyrr.), 7.87 (d, *J =* 1.9 Hz, 4H, H_o-Ar_), 7.74 (t, *J =* 1.9 Hz, 2H, H_p-Ar_), 7.15 (m, 4H, H_a_), 6.75 (br, 4H, H_b_), 6.50 (br, 4H, H_c_), 5.80 (br, 4H, H_d_), 1.47 (s, 36 H, CH_3_). MS (Maldi-TOF, dithranol): Calcd for [C_72_H_66_N_6_NiS_2_]^+•^: 1136.41; found 1136.40. UV-Vis. (CH_2_Cl_2_): λ_max_ = 414 nm (ε = 248,000 M^−1^.cm^−1^), 533 (19,000), 566 (11,000). Anal. Calcd for C_72_H_66_N_6_NiS_2_: C 75.98, H 5.84, N 7.38; found C 75.70, H 5.90, N 7.32. Crystal data. From CH_2_Cl_2_-CH_3_OH, C_74_H_70_Cl_4_N_6_NiS_2_, M = 1307.99 g.mol^−1^, 0.25 × 0.20 × 0.10 red prisms, triclinic, space group P-1, a = 9.581(5) Å, b = 12.043(5) Å, c = 15.226(5) Å, α = 69.453° (5), β = 84.502° (5), γ = 83.241° (5), V = 1630.8 (12) Å^3^, Z = 1, T = 173 K, MoKα = 0.71073, 1.81 < θ < 30.05, 35,851 reflections measured, 9530 unique reflections, R_1_ = 0.0579, wR_2_ = 0.1505, GoF = 1.033. CCDC Nr 2261554.

**Monophenoxazine 2**. The preparation and characterization of this compound were described previously [17]. Crystal data. C_53_H_37_N_5_NiO_2_, M = 834.58 g.mol^−1^, 0.500 × 0.380 × 0.160 mm red, triclinic, space group P-1, a = 9.3928 (6) Å, b = 16.1279 (11) Å, c = 16.7580 (11) Å, α = 74.871° (2), β = 83.3960° (10), γ = 87.470° (2), V = 2434.1 (3) Å^3^, Z = 2, T = 173 K, MoKα = 0.71073, 1.308 < θ < 28.125, 56,323 reflections measured, 11,860 unique reflections, R_1_ = 0.0515, wR_2_ = 0.1166, GoF = 1.048. CCDC Nr 2261556.

**Compound 3.** A Schlenk tube filled with bis(4-methoxyphenyl)-amine (298 mg, 1.3 mmol), tBuOK (1.5 g, 13.5 mmol), Pd(OAc)_2_ (14 mg, 0.06 mmol), rac-BINAP (58 mg, 0.09 mmol), and 18-crown-6 (15 mg, 0.06 mmol) was dried under vacuum for two hours and backfilled with argon. A degassed solution of 5-iodo-10,20-ditolyl-15-(*p*-anisyl)-nickel(II) porphyrin (100 mg, 0.13 mmol) in freshly distilled THF (60 mL) was added to the previously dried solids, and the reaction mixture was stirred at 70 °C under argon. After 16 h, the mixture was cooled to room temperature and the solvent evaporated. Chromatographic purification (silica gel, CH_2_Cl_2_/C_6_H_12_ 3/7) followed by crystallization (CH_2_Cl_2_, MeOH) afforded the dehalogenated porphyrin (30 mg, 35%) and the desired product **3** (35 mg, 0.04 mmol, 31%). ^1^H NMR (500 MHz, CDCl_3_, 25 °C), δ (ppm) = 9.04 (d, *J =* 5.0 Hz, 2H, pyrr.), 8.67 (d, *J =* 5.0 Hz, 2H, pyrr.), 8.66 (d, *J =* 5.0 Hz, 2H, pyrr.), 8.64 (d, *J =* 5.0 Hz, 2H, pyrr.), 7.87 (d, *J =* 6.7 Hz, 2H, H_o-Anis_), 7.82 (d, *J =* 8.0 Hz, 4H, H_o-Tolyl_), 7.42 (d, *J =* 8.0 Hz, 4H, H_m-Tolyl_), 7.18 (d, *J =* 6.7 Hz, 2H, H_m-Anis_), 7.12 (d, *J =* 6.7 Hz, 8H, H_N-Anis_), 6.70 (d, *J =* 6.7 Hz, 8H, H_N-Anis_), 4.02 (s, 4H, OCH_3_), 3.68 (s, 6H, OCH_3_), 2.60 (s, 6H, CH_3_). ^13^C (125 MHz, CDCl_3_): δ (ppm) = 159.3, 153.7, 164.1, 145.2, 143.2, 143.1, 142.0, 137.7, 137.4, 134.7 (CH), 133.6 (CH), 133.2, 133.1 (CH), 132.1 (CH), 132.0 (CH), 130.8 (CH), 127.6 (CH), 123.0 (CH), 122.0, 119.1, 119.0, 114.5 (CH), 112.4 (CH), 55.6 (OCH_3_), 55.5 (OCH_3_), 21.5 (CH_3_). UV-Vis. (CH_2_Cl_2_): λ_max_ = 410 nm (ε = 152,000 M^−1^.cm^−1^), 470 (28,000sh), 541 (12,800), 595 (7000). Anal. Calcd for C_55_H_43_N_5_NiO_3_: C 75.01, H 4.92, N 7.95; found C 74.62, H 4.93, N 7.76.

**Compound 6.** A Schlenk tube filled with bis(4-methoxyphenyl)-amine (415 mg, 1.80 mmol), tBuOK (2.0 g, 18 mmol), Pd(OAc)_2_ (21 mg, 0.09 mmol), rac-BINAP (78.5 mg, 0.13 mmol), and 18-crown-6 (20 mg, 0.08 mmol) was dried under vacuum for one hour and backfilled with argon. A degassed solution of 5,15-diodo-10,20-di-(3,5-di-tBu-phenyl)-nickel(II) porphyrin (180 mg, 0.18 mmol) in freshly distilled THF (60 mL) was added to the previously dried solids, and the reaction mixture was stirred at 70 °C under argon. After 15 h, the mixture was cooled to room temperature and the solvent evaporated. Chromatographic purification (silica gel, CH_2_Cl_2_/C_6_H_12_ 3/7) followed by crystallization (CH_2_Cl_2_, n-hexane, MeOH) afforded green crystals (49 mg, 0.041 mmol, 23%). ^1^H NMR (500 MHz, CDCl_3_, 25 °C), δ (ppm) = 9.00 (d, *J =* 5.0 Hz, 4H, pyrr.), 8.59 (d, *J =* 5.0 Hz, 4H, pyrr.), 7.75 (d, *J =* 1.7 Hz, 4H, H_o-Ar_), 7.65 (t, *J =* 1.7 Hz, 2H, H_p-Ar_), 7.12 (d, *J =* 9.1 Hz, 8H, H_Anis_), 6.71 (d, *J =* 9.1 Hz, 8H, H_Anis_), 3.68 (s, 12H, OCH_3_), 1.42 (s, 36H, CH_3_). ^13^C (125 MHz, CDCl_3_): δ (ppm) = 153.6, 148.9, 146.0, 145.3, 142.4, 139.5, 133.4 (CH), 130.6 (CH), 128.4 (CH), 122.9 (CH), 122.2, 121.3 (CH), 120.3, 114.5 (CH), 55.5 (OCH_3_), 35.0, 31.7 (CH_3_). MS (ESI-TOF-HRMS): Calcd for [C_76_H_78_N_6_NiO_4_]^+•^: 1196.5433; found 1196.5439. UV-Vis. (CH_2_Cl_2_): λ_max_ = 401 nm (ε = 115,000 M^−1^.cm^−1^), 477 (53,000), 545 (10,500), 622 (11,300). Anal. Calcd for C_76_H_78_N_6_NiO_4_•H_2_O: C 75.06, H 6.63, N 6.91; found C 75.11, H 6.48, N 6.93. Crystal data. From chlorobenzene and isopropanol, C_76_H_78_Cl_4_N_6_NiO_4_, M = 1198.15 g.mol^−1^, 0.500 × 0.500 × 0.500 red, triclinic, space group P-1, a = 10.7094 (10) Å, b = 14.9585 (16) Å, c = 20.634 (2) Å, α = 97.122° (5), β = 90.811° (5), γ = 99.452° (6), V = 3233.4 (6) Å^3^, Z = 2, T = 173 K, CuKα = 1.54178, 3.932 < θ < 66.975, 44,205 reflections measured, 11,200 unique reflections, R_1_ = 0.088, wR_2_ = 0.2262, GoF = 1.030. CCDC Nr 2261547.

### 3.3. Electrochemistry

Electrochemical measurements were carried out in CH_2_Cl_2_ containing NBu_4_PF_6_ (0.1 M) in a classical three-electrode cell by cyclic voltammetry (CV) and rotating-disk voltammetry (RDV). The working electrode was a glassy carbon disk (3 mm in diameter), the auxiliary electrode was a Pt wire, and the pseudo-reference electrode was a Pt wire. The cell was connected to an Autolab PGSTAT30 potentiostat (Eco Chemie, Utrecht, Holland) driven by GPSE software running on a personal computer. All potentials are given vs. Fc^+^/Fc used as internal standards and are uncorrected from ohmic drop. Spectroelectrochemical investigations were carried out with a MCS600 Carl Zeiss photodiodes array spectrometer (MCS 601 UV-vis and MCS 611 NIR2.2 modules) controlled by the Aspect Plus software. A home-made Optical Transparent Thin Layer Electrode (OTTLE) spectroelectrochemical cell was placed in the light beam. The three electrodes of the cell (a platinum grid as the working electrode, platinum wire as the counter electrode, and silver/silver chloride as the reference electrode) were connected to an Autolab 302 potentiostat/galvanostat controlled by the GPES software running on a PC computer.

### 3.4. EPR Measurements

CW EPR experiments were performed using an X-band (9–10 GHz) Bruker ESP300 spectrometer equipped with a standard rectangular cavity (TE102). A Bruker Elexis E580 spectrometer operating at the X-band (9–10 GHz) equipped with an Oxford instrument CF935 cryostat, an ITC4 temperature controller, and a 1 kW TWT amplifier was used for pulsed EPR measurements. 2D HYSCORE (hyperfine sublevel correlation spectroscopy) spectra were measured by using a pulse sequence (π/2–τ–π/2–t1–π-t2–π/2–τ echo), with pulse lengths of 16 and 32 ns for the π/2 and π pulses, respectively. The experiments were performed with two different delay values τ (136 and 200 ns). Numerous unwanted pulse echoes were removed by four-step phase cycling. The intensity of the echo was measured after the fourth pulse with variable t1 and t2 values, keeping τ constant. The collected ESEEM and HYSCORE data were Fourier transformed by using the spectrum manipulation routines available within the Bruker program: subtraction of the relaxation decay, apodization (Hamming window), and zero filling. The HYSCORE spectra were recorded with a magnetic field corresponding to the maximum intensity of the radical signal in the two-pulse field-swept EPR spectra. The EPR and HYSCORE spectra were simulated using Easyspin V5.2.28 using the MATLAB R2020a version [29].

### 3.5. Computational Methods

The ORCA program package was used to perform all theoretical calculations based on density functional theory (DFT) [30]. Starting from the experimentally determined solid-state structures, all complexes were subjected to full geometry optimization using the B3LYP functional [31,32] in combination with the 6–31 g* basis sets [33,34,35]. Increased integration grids and tight SCF convergence criteria were used in the calculations. Cartesian coordinates of the DFT-optimized structures are provided in the SI. Solvent effects were accounted for according to the experimental conditions, and dichloromethane was used as a solvent (ε = 80) within the framework of the conductor-like polarizable continuum model, COSMO [36]. Single point calculations were conducted to compute electronic structures and predict EPR parameters using the B3LYP functional together with the CP(PPP) basis set for the metal center [37] and the EPR-II basis set for other atoms [38]. Optimized geometries as well as electronic structures were visualized using the program Chemdraft.

## 4. Conclusions

This work demonstrates that the introduction of aromatic amines on the free *meso* positions of diarylporphyrins via either Buchwald or Ullmann methods is very efficient. Radicals can be generated on the nitrogen atoms by electrochemistry and chemical oxidation. The degree of communication between the radicals through the π-electron density of the porphyrin ring strongly depends on the aromatic amines’ ability to contribute to their delocalization. The higher global flexibility of amine substituents able to freely rotate along both the N-C*_meso_* and the N-Aryl bonds allows a more efficient delocalization of the radicals.

## Figures and Tables

**Table 1:** Electrochemical data for the six compounds ^a^.

Compound	E_red2_ (Volts)	E_red1_ (Volts)	E_ox1_ (Volts)	ΔEp (mV)	E_ox2_ (Volts)	ΔEp (mV)	E_ox3_ (Volts)	E_ox4_ (Volts)
**1**	−2.18 ^b^	−1.64 (1)	+0.34 (1)	60	+0.85 (2)	100	+1.24 ^b^	-
**4**	−2.20 ^b^	−1.62 (1)	+0.34 (1)	60	+0.40 (1)	60	+0.98 (2)	+1.25 ^b^
**2**	-	−1.70 (1)	+0.36 (1)	80	+0.84 (2)	100	-	-
**5**	-	−1.64 (1)	+0.37 (1)	60	+0.43 (1)	75	+0.99 (2)	-
**3**	-	−1.09 ^b^	+0.58 (1)	133	+0.95 (2)	122	-	-
**6**	-	−1.09 ^b^	+0.57 (2)	100	+1.06 ^b^	-	-	-

^a^. Cyclic voltammetry measurements: 1 mM dichloromethane solutions, with NBu_4_PF_6_ (0.1 M) as the supporting electrolyte, scan rate 0.1 V/s, 298 K. The number of exchanged electrons (indicated in parentheses) was determined by rotating disk voltammetry (RDV). Potentials are referenced vs. the Fc+/Fc redox couple, except for compounds **3** and **6** measured with the AgCl/Ag reference electrode. ^b^. Irreversible reduction or oxidation steps.

**Table 2:** Isotropic hyperfine coupling constants obtained from DfT computations compared to experimental values from CW-EPR and ENDOR spectra (values in MHz).

Compound		N	H_1_	H_2_	H_3_	H_4_	g_iso_
**1**	Exp	19	-	5.6	2.0	2.2	2.0034
	DFT	15	1.2	6.2	2.2	2.3	2.0060
**2**	Exp	23	1.3	8.3	2.1	4.3	2.0035
	DFT	18	1.9	8.2	2.0	3.3	2.0037
**3**	Exp	19	4.3	1.8	-	-	2.0035
	DFT	15	4.9	1.5	-	-	2.0033

**Table 3:** ^14^N hyperfine and quadrupolar coupling parameters obtained from DFT computations and experimental HYSCORE spectra (values in MHz) ^a^.

	Compound 1				Compound 2				Compound 3			
	Exp	DFT	Exp	DFT	Exp	DFT	Exp	DFT	Exp	DFT	Exp	DFT
	^14^N_porphyrin_		^14^N_phenothiazine_		^14^N_porphyrin_		^14^N_phenoxazine_		^14^N_porphyrin_		^14^N_anisyl_	
T (MHz) ± 0.1	0.2	0.1 ^b^	14 ^a^	14	0.1	0.1 ^b^	13 ^a^	17 ^a^	0.5	1.1 ^c^	-	12
|A_iso_| (MHz) ± 0.1	0.6	0.35 ^b^	19	15	0.8	0.5 ^b^	19 ^c^	18	1.2	1.2 ^c^	-	19
|e^2^qQ/h| (MHz) ± 0.1	1.8	2.1 ^b^	3.5	3.2	1.8	2.2 ^b^	2.8	2.8	2.0	2.0 ^c^	-	3.6
η ± 0.1	0.2	0.1 ^b^	0.1	0.1	0.2	0.13 ^b^	0.1	0.0	0.1	0.1 ^c^	-	0

^a^ Values are in agreement with hyperfine coupling originating from field-swept EPR spectra (see Figure S5 and Table S1 in the Supporting Information). ^b^ Average value considering the two porphyrin nitrogen atoms featuring some spin density. ^c^ Average value for the four porphyrin nitrogen atoms. Simulations are shown in Figures S14 and S15 in SI. T is the dipolar contribution, e^2^qQ/h is the quadrupolar coupling constant, and η is the electric field gradient asymmetry parameter.

## Supplementary Materials

The following supporting information can be downloaded at: https://www.mdpi.com/article/10.3390/molecules28114405/s1, Figure S1: ^1^H NMR spectrum of compound **1**; Figure S2: MALDI-TOF MS of **1** (top) simulation (bottom); Figure S3: ^1^H NMR spectrum of compound **4**; Figure S4: MALDI-TOF MS of **4** (top) simulation (bottom); Figure S5: ^1^H NMR of compound **3** (top) and aromatic area (bottom); Figure S6: ^13^C NMR of compound **3** (top) and DEPT (bottom); Figure S7: ^1^H NMR of compound **6**; Figure S8: HRMS (ESI-TOF) of compound **6** (top) and simulation (bottom); Figure S9: ^13^C NMR of compound **6** (top) and DEPT (bottom); Figure S10: UV/Vis spectra during the stepwise electrochemical oxidation of **2**: (a) at the first oxidation potential; (b) at the second oxidation potential (recorded in dichloromethane, 0.1 M NBu_4_PF_6_); Figure S11: (a) X-band EPR spectrum of **2** in CH_2_Cl_2_ fluid solution at room temperature (blue) and its simulation (red) (b) ^1^H-ENDOR spectrum of **2** in CH_2_Cl_2_ fluid solution at 200K; Figure S12: (a) X-band EPR spectrum of **3** in CH_2_Cl_2_ fluid solution at room temperature (b) ^1^H-ENDOR spectrum of **3** in CH_2_Cl_2_ fluid solution at 200 K; Figure S13: (++) and (+ −) quadrants of the ^1^H and ^14^N of X-band HYSCORE spectrum at 80 K of (a) **2** (b) **3** showing the location of ^14^N cross-peaks weakly coupled nitrogen nuclei, respectively, and ^1^H cross-ridges. Microwave frequency of 9.71 and 9.74 GHz respectively, magnetic field 350.0 and 352.5 mT respectively and time τ of 136 ns; Figure S14: Experimental (blue) and simulated (red) X-band ^14^N–HYSCORE spectra of (a) **1**, (b) **2** and (c) **3.** The simulations are carried out using parameters given in Table 2 in the main text; Figure S15: Field-swept EPR spectra at 80 K of (a) **1** (b) **2** and (c) **3.** Experimental (black) and simulated (red); Figure S16: Experimental EPR spectrum of (a) **5** and (b) **6** in CH_2_Cl_2_ fluid solution at room temperature; Figure S17: Experimental EPR spectrum of mixture **2** and **4** in CH_2_Cl_2_ fluid solution generated by electrolysis at room temperature. Simulated spectrum was obtained by an admixture of 23% of monoradical and 77% of biradical; Figure S18: Temperature dependence of the EPR susceptibility (χT product) in CH_2_Cl_2_ frozen solution for (a) **5** and (b) **4**; Table S1: ^14^N hyperfine coupling parameters in MHz obtained from simulations and experimental Field-swept EPR spectra; and DFT calculations for compounds **1**, **2**, and **3**.

## Sample Availability

Samples of all compounds are available from the authors.
