# Supplementary material for: Syntheses and Electrochemical and EPR Studies of Porphyrins Functionalized with Bulky Aromatic Amine Donors
Source: Molecules. 2023 May 29;28(11):4405. doi: 10.3390/molecules28114405 (PMC10254424; doi:10.3390/molecules28114405)
Supplement: Supplementary file 1 [file molecules-28-04405-s001.zip › molecules-2418169-supplementary.pdf]

Syntheses, electrochemical and EPR studies of porphyrins functionalized with bulky aromatic amine donors

Mary-Ambre Carvalho[1], Khalissa Merahi[1], Julien Haumesser[1], Ana M. V. M. Pereira[1], Nathalie Parizel[1], Jean Weiss[1], Maylis Oro[2], Vincent Maurel[3], Laurent Ruhlmann[1,*], Sylvie Choua[1,*] and Romain Ruppert[1,*]

Supporting information

Table of contents:

Figure S1: ^{1}H NMR spectrum of compound **1** 2

Figure S2: MALDI-TOF MS of **1** (top) simulation (bottom) 3

Figure S3: ^{1}H NMR spectrum of compound **4** 4

Figure S4: MALDI-TOF MS of **4** (top) simulation (bottom) 5

Figure S5: ^{1}H NMR of compound **3** (top) and aromatic area (bottom). 6

Figure S6: ^{13}C NMR of compound **3** (top) and DEPT (bottom). 7

Figure S7: ^{1}H NMR of compound **6**. 8

Figure S8: HRMS (ESI-TOF) of compound **6** (top) and simulation (bottom). 8

Figure S9: ^{13}C NMR of compound **6** (top) and DEPT (bottom). 9

Figure S10: UV/Vis spectra during the stepwise electrochemical oxidation of **2**: (a) at the first oxidation potential; (b) at the second oxidation potential (recorded in dichloromethane, 0.1 M NBu_4PF_6). 10

Figure S11: a) X-band EPR spectrum of **2** in CH_2Cl_2 fluid solution at room temperature (blue) and its simulation (red) b) ^{1}H-ENDOR spectrum of **2** in CH_2Cl_2 fluid solution at 200K. 11

Figure S12: a) X-band EPR spectrum of **3** in CH_2Cl_2 fluid solution at room temperature b) ^{1}H-ENDOR spectrum of **3** in CH_2Cl_2 fluid solution at 200 K. 11

Figure S13: (++) and (+ −) quadrants of the ^{1}H and ^{14}N of X-band HYSCORE spectrum at 80 K of a) **2** b) **3** showing the location of ^{14}N cross-peaks weakly coupled nitrogen nuclei, respectively, and ^{1}H cross-ridges. Microwave frequency of 9.71 and 9.74 GHz respectively, magnetic field 350.0 and 352.5 mT respectively and time τ of 136 ns. 12

Figure S14: Experimental (blue) and simulated (red) X-band ^{14}N–HYSCORE spectra of a) **1**, b) **2** and c) **3.** The simulations are carried out using parameters given in Table 2 in the main text. 13

Figure S15: Field-swept EPR spectra at 80 K of a) **1** b) **2** and c) **3.** Experimental (black) and simulated (red). 14

Table S1: ^{14}N hyperfine coupling parameters in MHz obtained from simulations and experimental Field-swept EPR spectra. 14

Figure S16: Experimental EPR spectrum of a) **5** and b) **6** in CH_2Cl_2 fluid solution at room temperature. 14

Figure S17: Experimental EPR spectrum of mixture **2** and **4** in CH_2Cl_2 fluid solution generated by electrolysis at room temperature. Simulated spectrum was obtained by an admixture of 23% of monoradical and 77% of biradical. 14

Figure S18: Temperature dependence of the EPR susceptibility (χT product) in CH_2Cl_2 frozen solution for a) **5** and b) **4**. 15

DFT calculations for compounds **1**, **2**, and **3**. 16

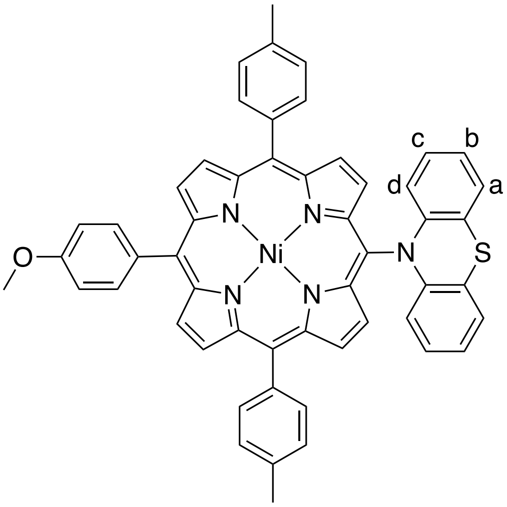

$C_{53}H_{37}N_5NiOS$
Exact Mass: 849,21
Molecular Weight: 850,67

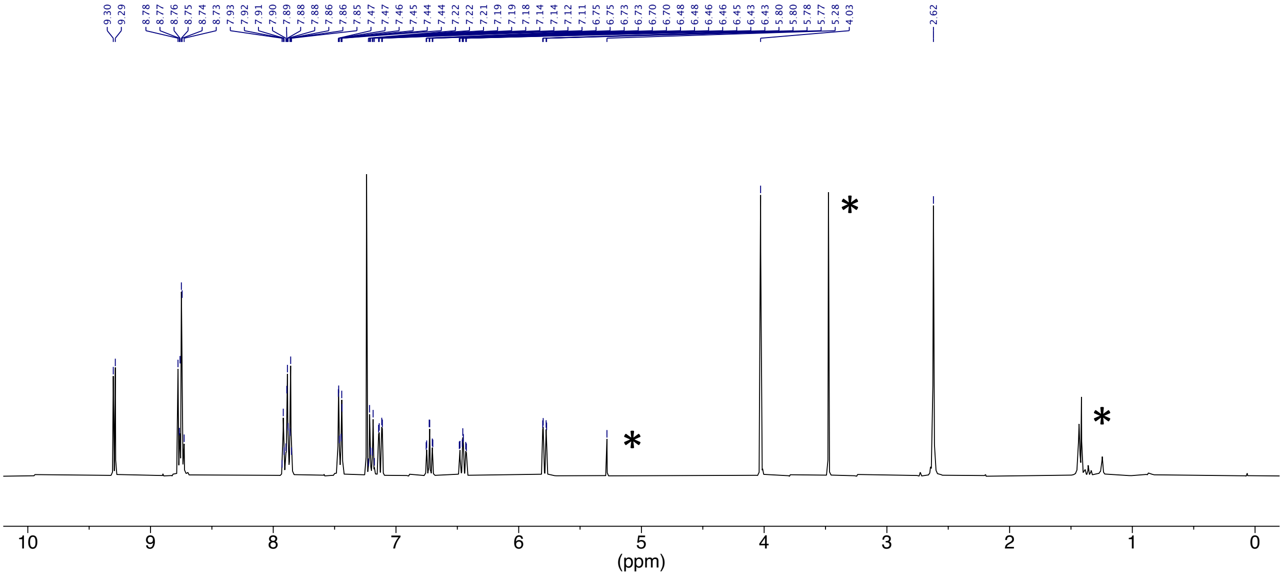

Figure S1: ^{1}H NMR of compound **1** (* residual solvent peaks, CH_2Cl_2, CH_3OH, C_6H_{12}, H_2O).

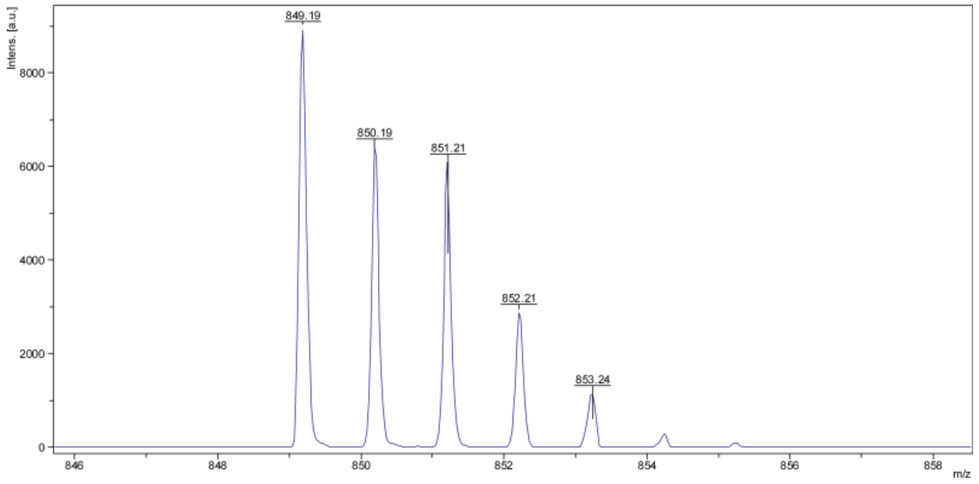

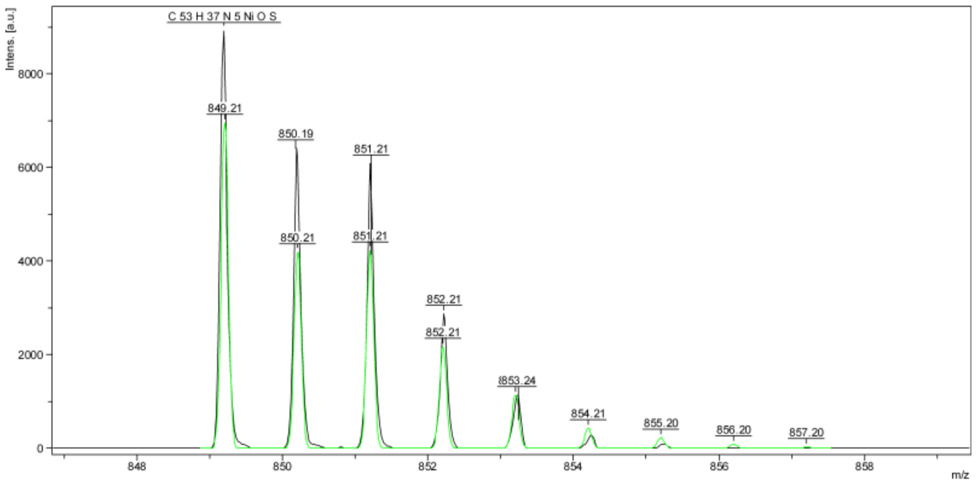

Figure S2: MS of compound **1**, Maldi-TOF (dithranol matrix)
experimental data (top) and experimental data + simulation (bottom)

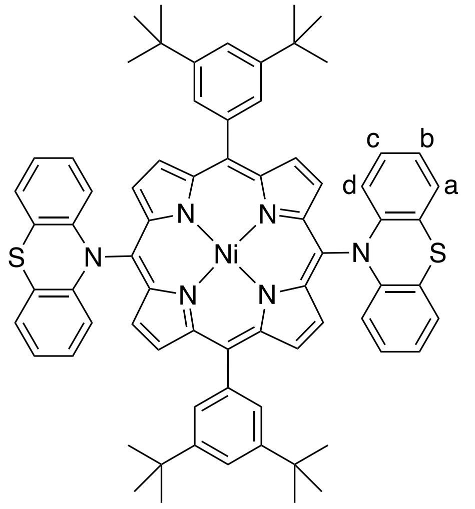

$C_{72}H_{66}N_6NiS_2$
Exact Mass: 1136,41
Molecular Weight: 1138,18

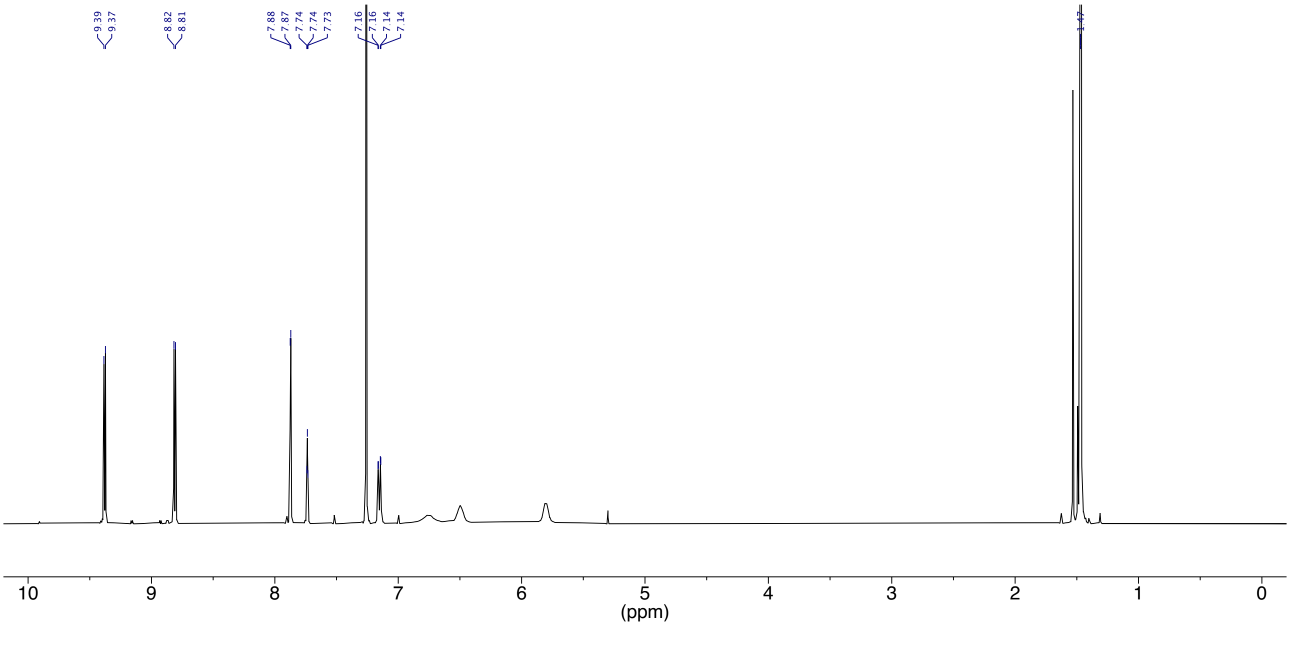

Figure S3: ^{1}H NMR of compound **4**

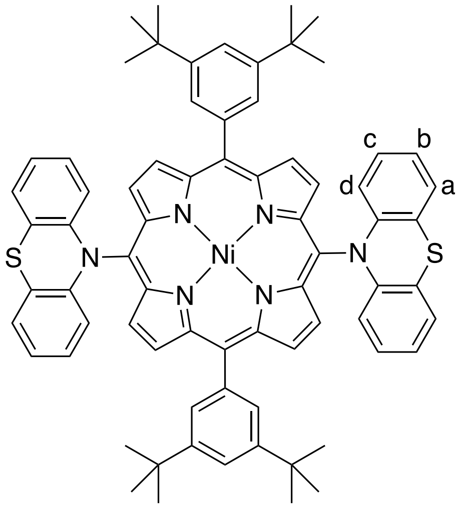

$C_{72}H_{66}N_6NiS_2$
Exact Mass: 1136,41
Molecular Weight: 1138,18

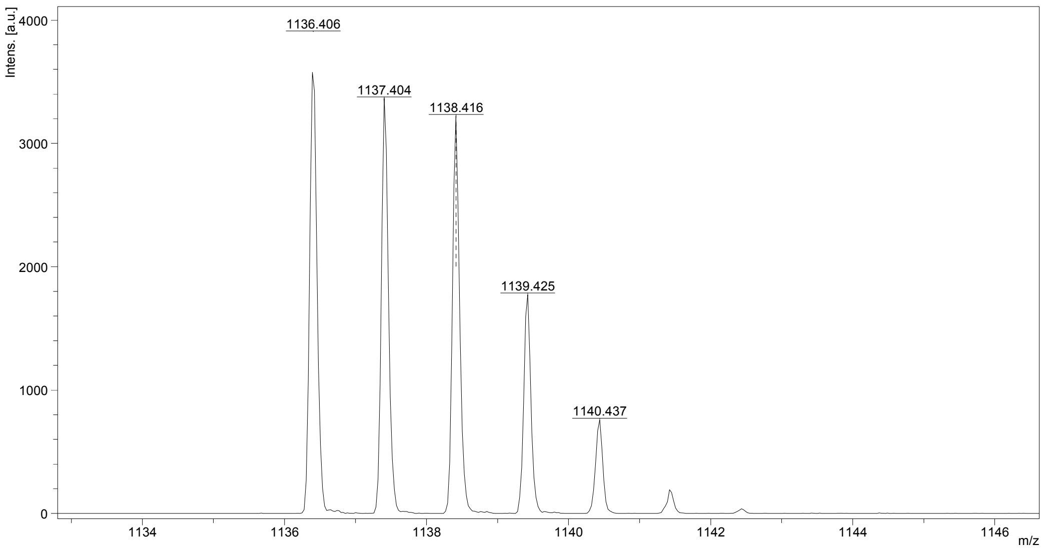

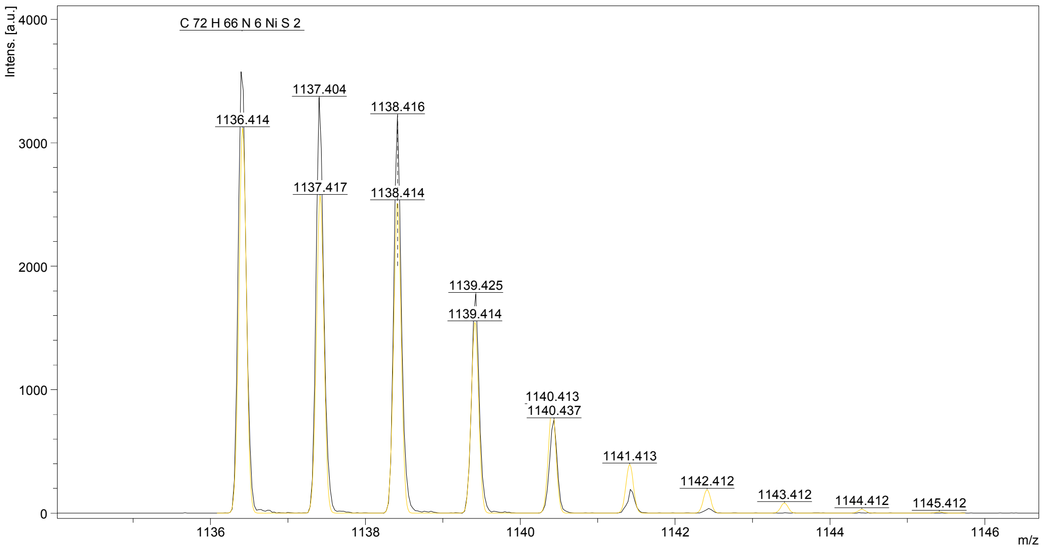

Figure S4: MS of compound **4**, Maldi-TOF (dithranol matrix)
experimental data (top) and experimental data + simulation (bottom)

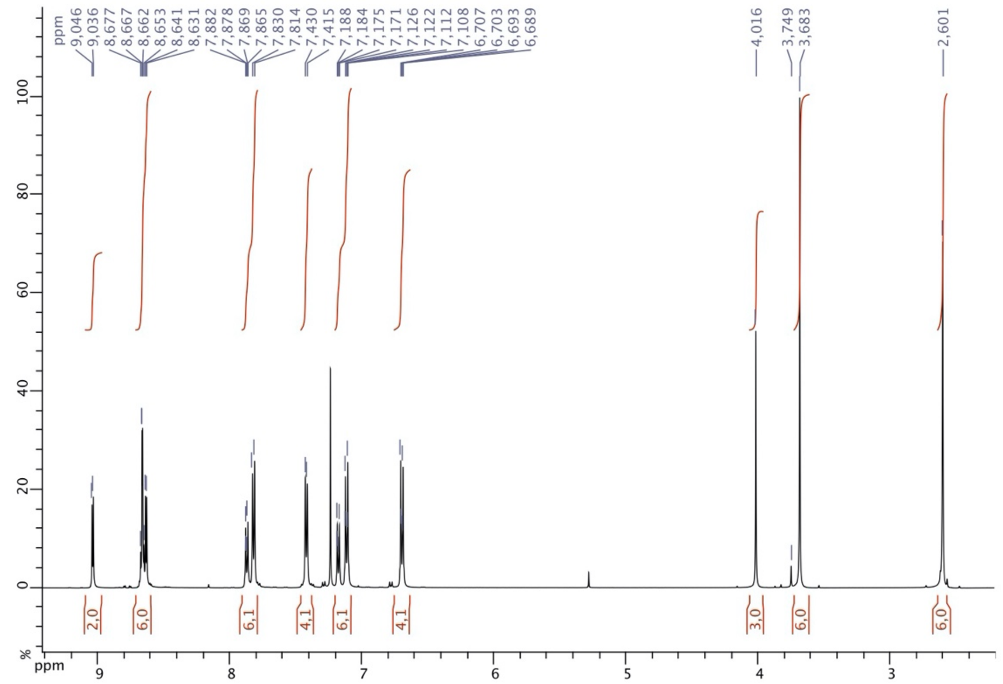

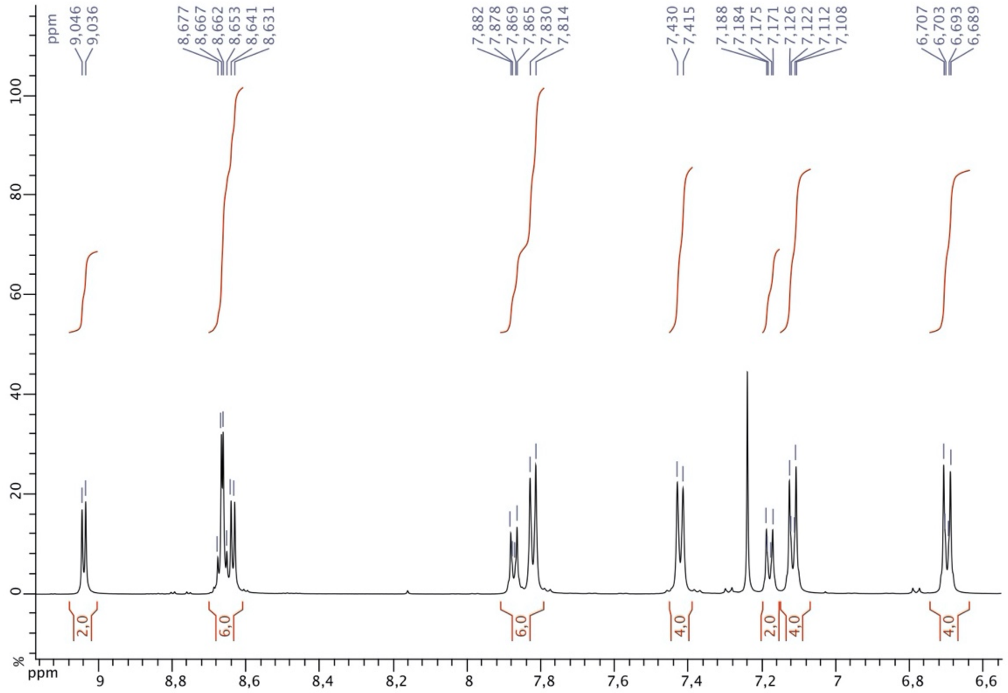

Figure S5: ^{1}H NMR of compound **3** (top) and aromatic area (bottom).

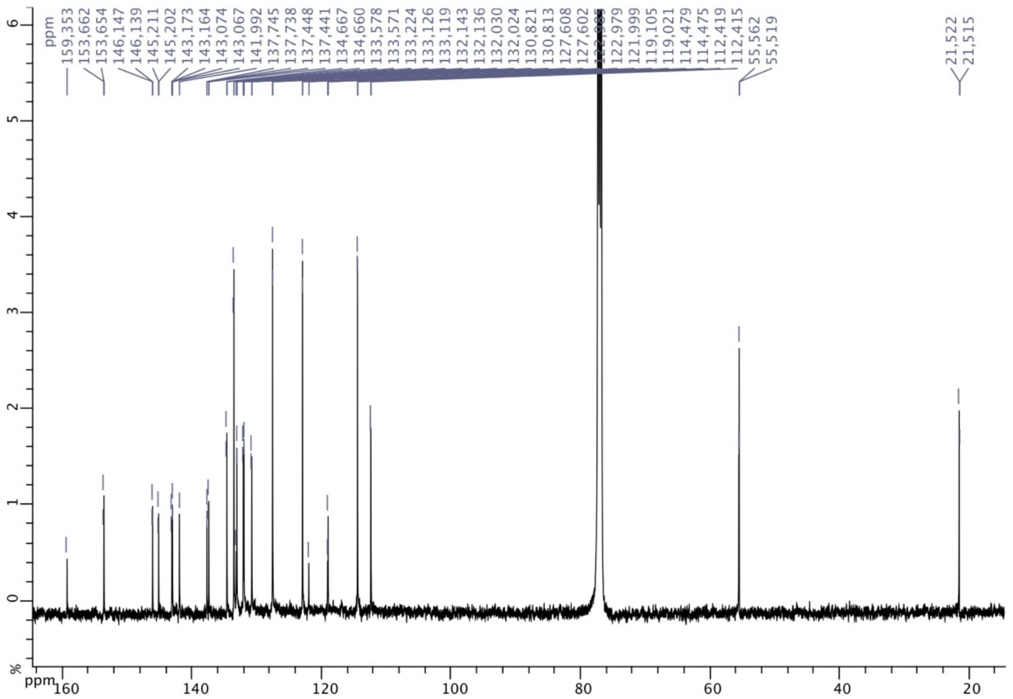

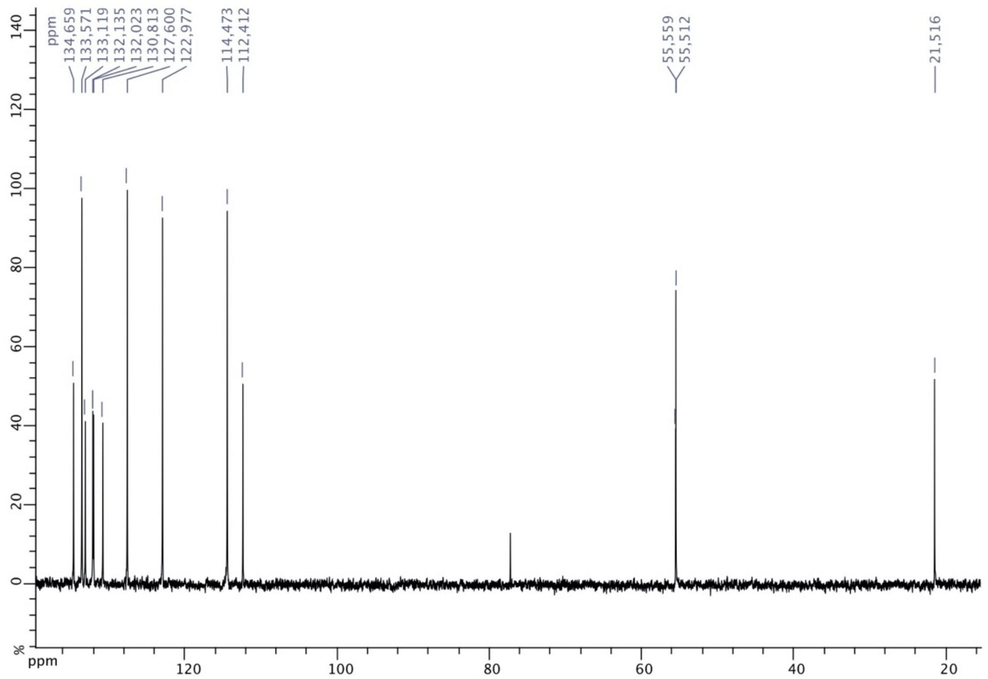

Figure S6: ^{13}C NMR of compound **3** (top) and DEPT (bottom).

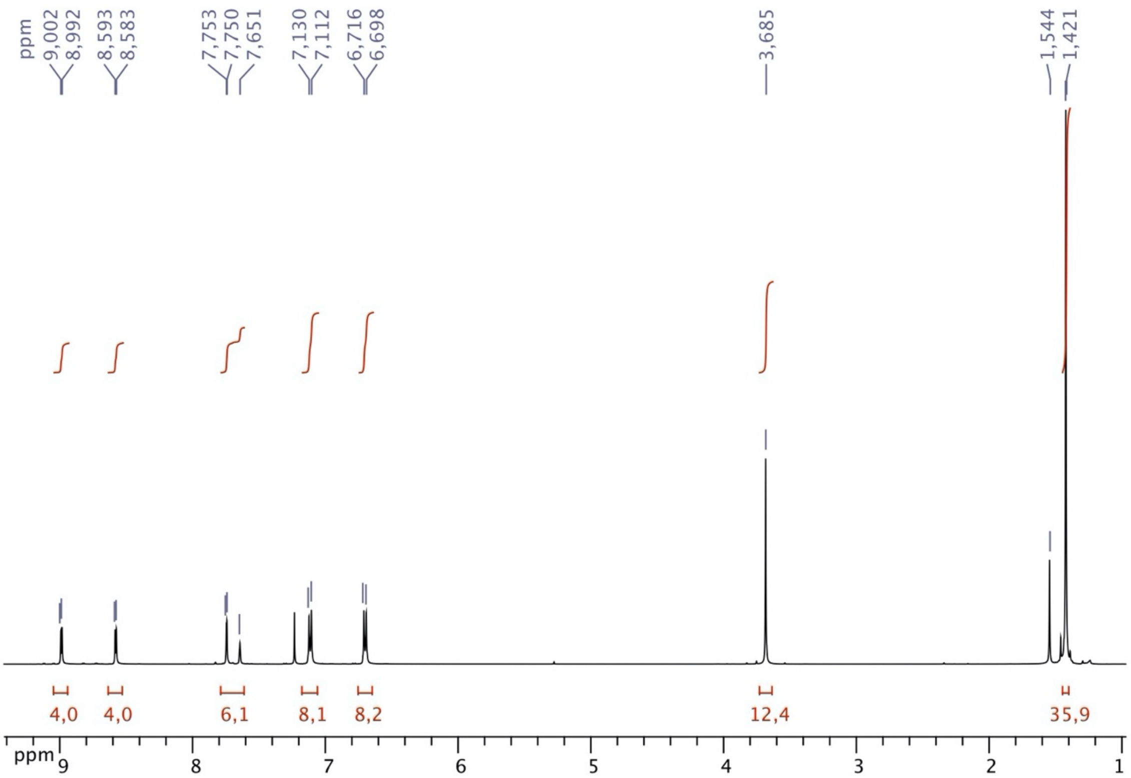

Figure S7: ^{1}H NMR of compound **6**.

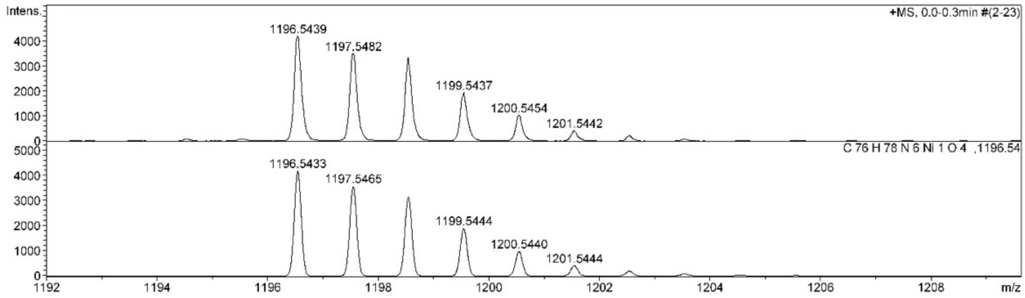

Sum Formula	Sigma	m/z	Err [ppm]	Mean Err [ppm]	rdb	N Rule	e⁻
C 76 H 78 N 6 Ni 1 O 4	0.02	1196.5433	-0.51	-0.63	41.00	-	odd
C 76 H 77 N 6 Ni 1 O 4	0.39	1195.5354	-4.36	-7.15	41.50	ok	even
C 76 H 76 N 6 Ni 1 O 4	0.55	1194.5276	-12.92	-15.18	42.00	-	odd

Figure S8: HRMS (ESI-TOF) of compound **6** (top) and simulation (bottom).

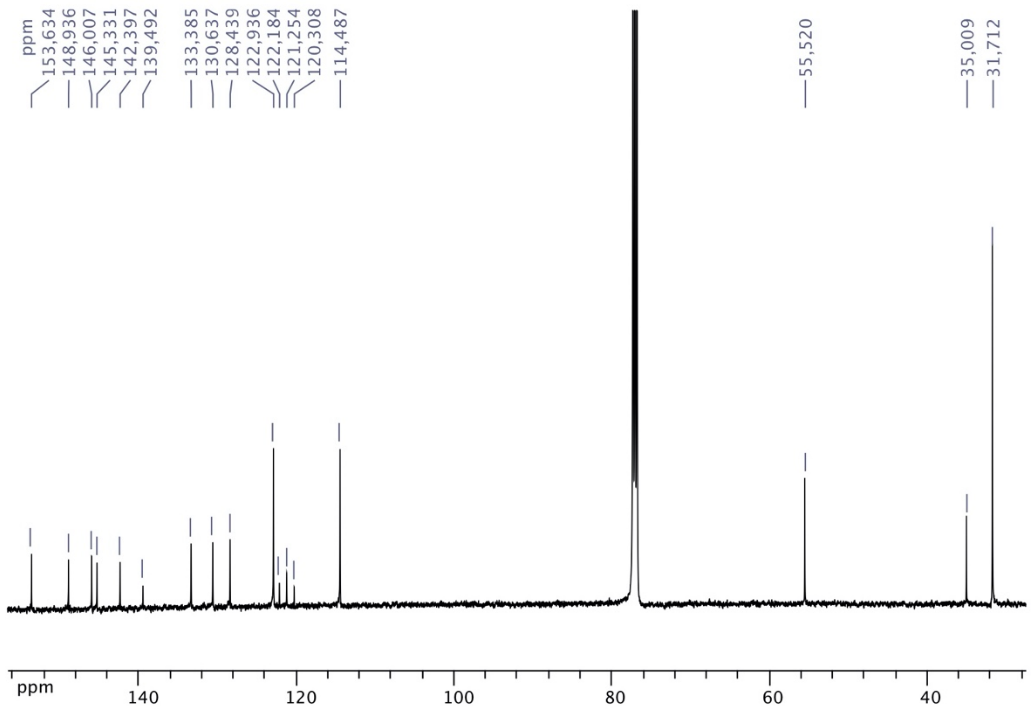

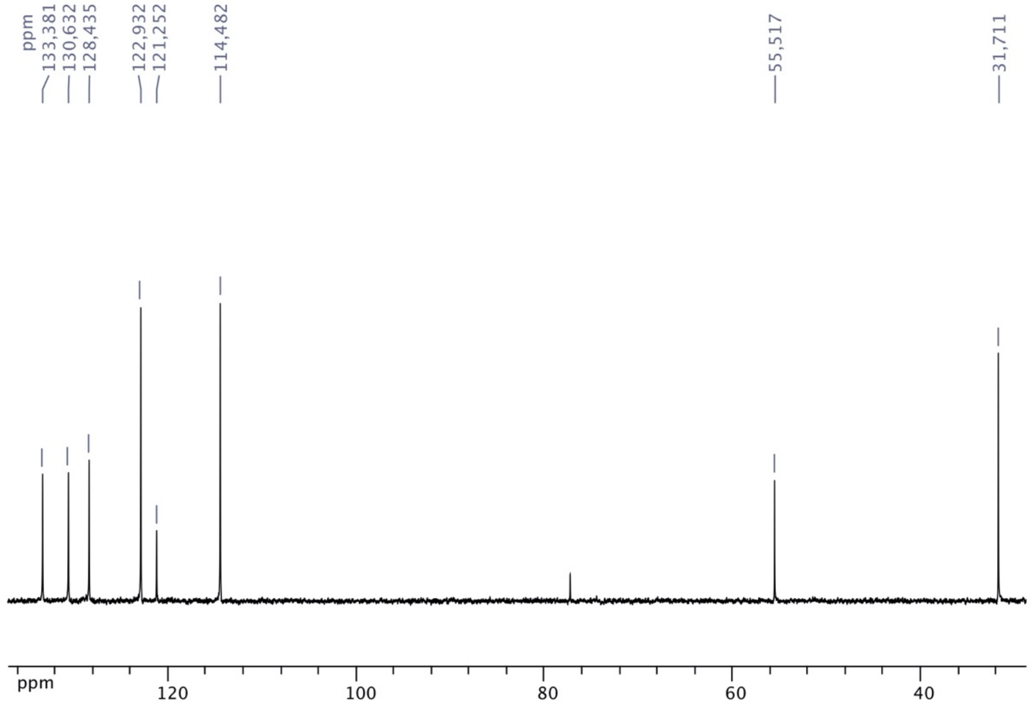

Figure S9: ^{13}C NMR of compound **6** (top) and DEPT (bottom).

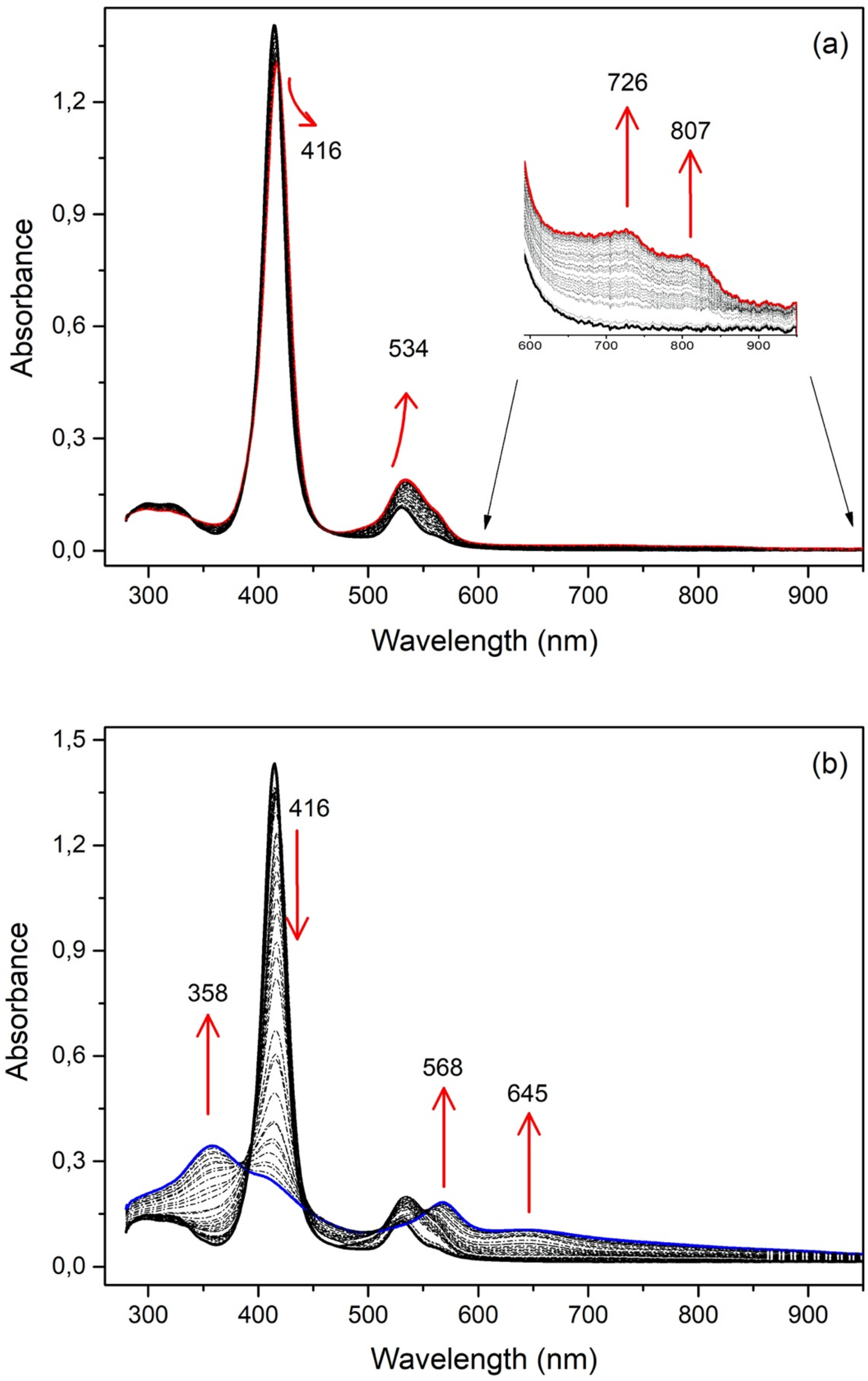

Figure S10: UV/Vis spectra during the stepwise electrochemical oxidation of **2**: (a) at the first oxidation potential; (b) at the second oxidation potential (recorded in dichloromethane, 0.1 M NBu_4PF_6).

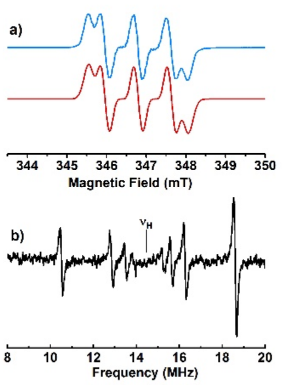

Figure S11: a) X-band EPR spectrum of **2** in CH_2Cl_2 fluid solution at room temperature (blue) and its simulation (red) b) ^{1}H-ENDOR spectrum of **2** in CH_2Cl_2 fluid solution at 200K.

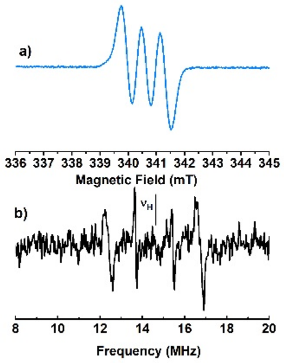

Figure S12: a) X-band EPR spectrum of **3** in CH_2Cl_2 fluid solution at room temperature b) ^{1}H-ENDOR spectrum of **3** in CH_2Cl_2 fluid solution at 200 K.

a)

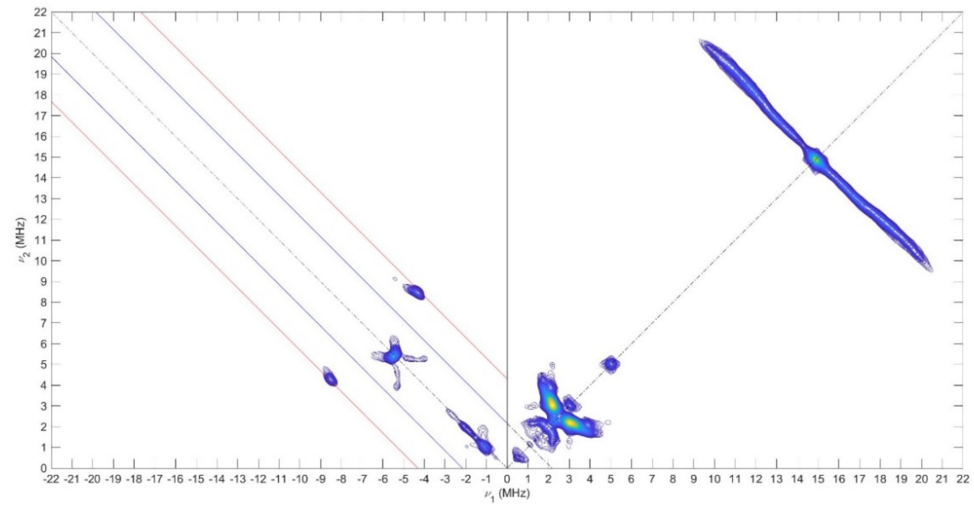

b)

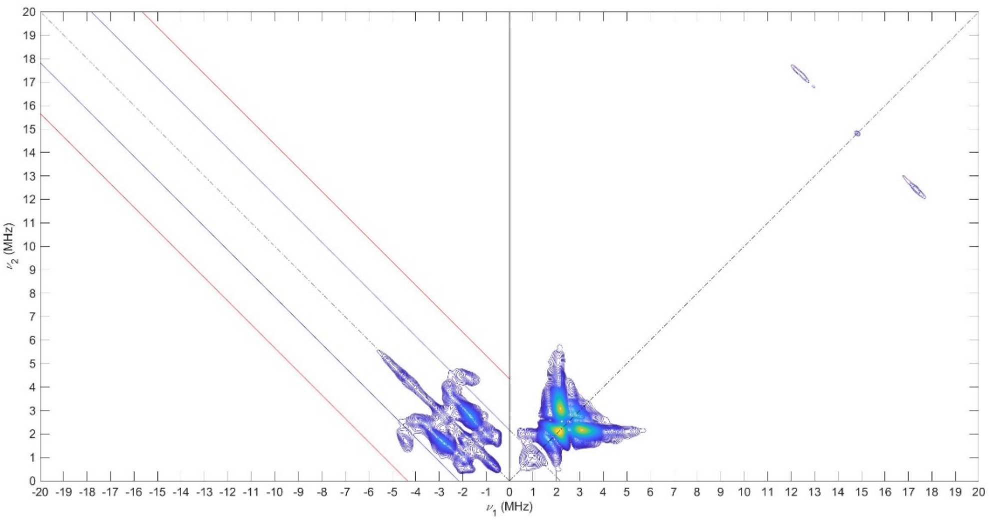

Figure S13: (++) and (+ −) quadrants of the ^{1}H and ^{14}N of X-band HYSCORE spectrum at 80 K of a) **2** b) **3** showing the location of ^{14}N cross-peaks weakly coupled nitrogen nuclei, respectively, and ^{1}H cross-ridges. Microwave frequency of 9.71 and 9.74 GHz respectively, magnetic field 350.0 and 352.5 mT respectively and time τ of 136 ns.

a)

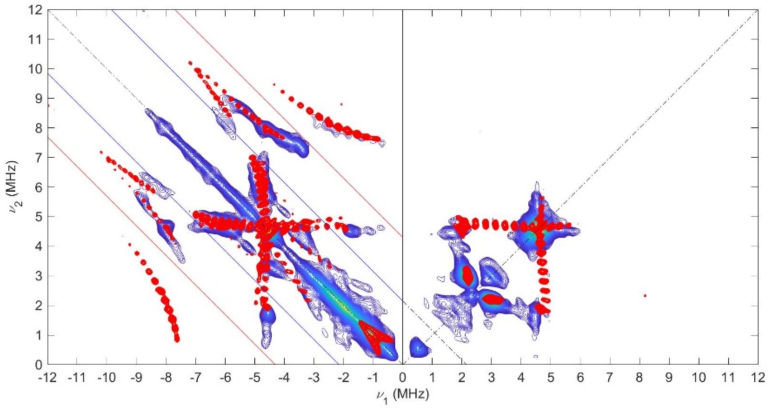

b)

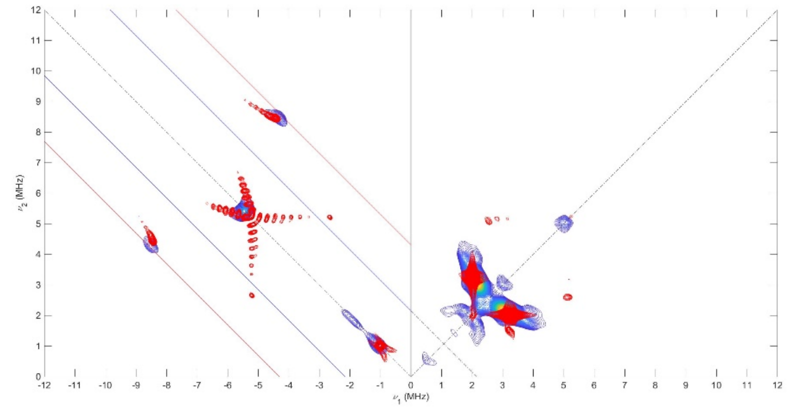

c)

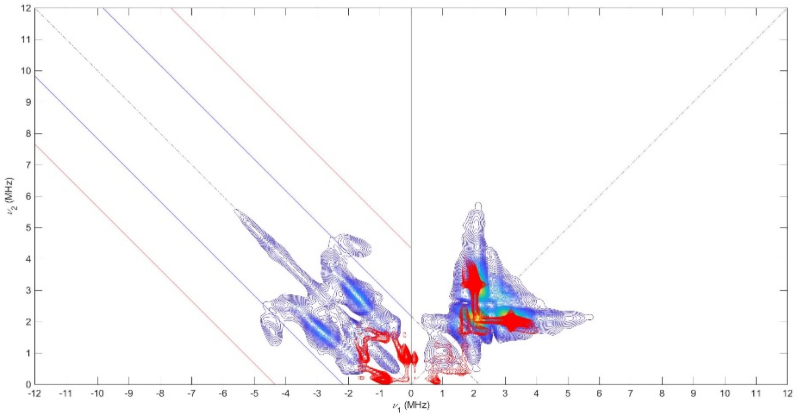

Figure S14: Experimental (blue) and simulated (red) X-band ^{14}N–HYSCORE spectra of a) **1**, b) **2** and c) **3.** The simulations are carried out using parameters given in Table 3 in the main text.

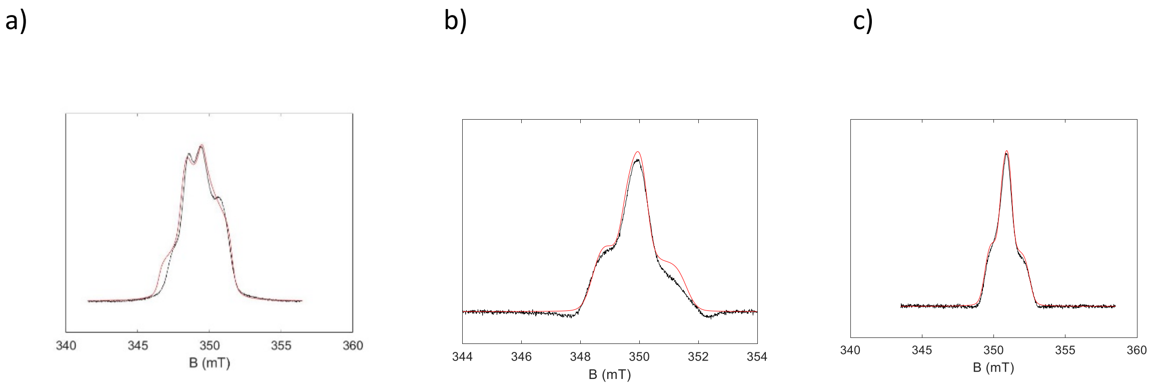

Figure S15: Field-swept EPR spectra at 80 K of a) **1** b) **2** and c) **3.** Experimental (black) and simulated (red).

**Table S1: ^{14}N hyperfine coupling parameters in MHz obtained from simulations and experimental Field-swept EPR spectra.**						
	**Exp**			**DFT**		
	A_x	A_y	A_z	A_x	A_y	A_z
**2**	2	2	44	1	1.3	51
**1**	1	1	42	1	1.1	43
**3**	2	2	44	1.3	1.3	42

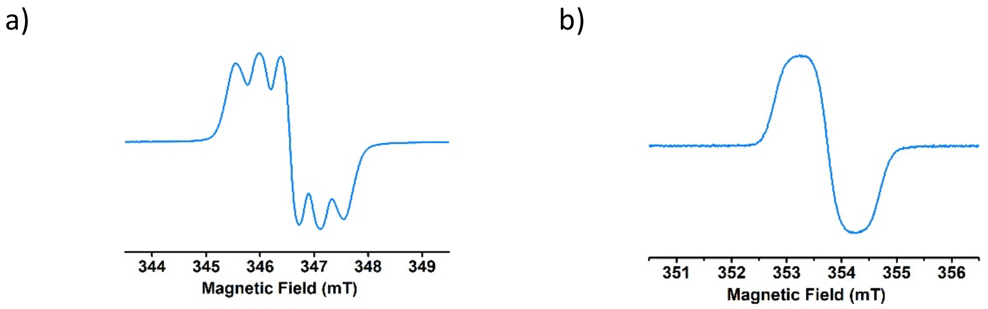

Figure S16: Experimental EPR spectrum of a) **5** and b) **6** in CH_2Cl_2 fluid solution at room temperature.

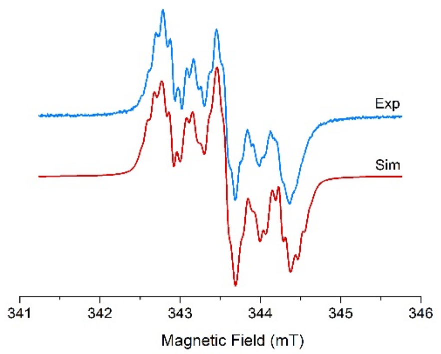

Figure S17: Experimental EPR spectrum of mixture **2** and **4** in CH_2Cl_2 fluid solution generated by electrolysis at room temperature. Simulated spectrum was obtained by an admixture of 23% of monoradical and 77% of biradical.

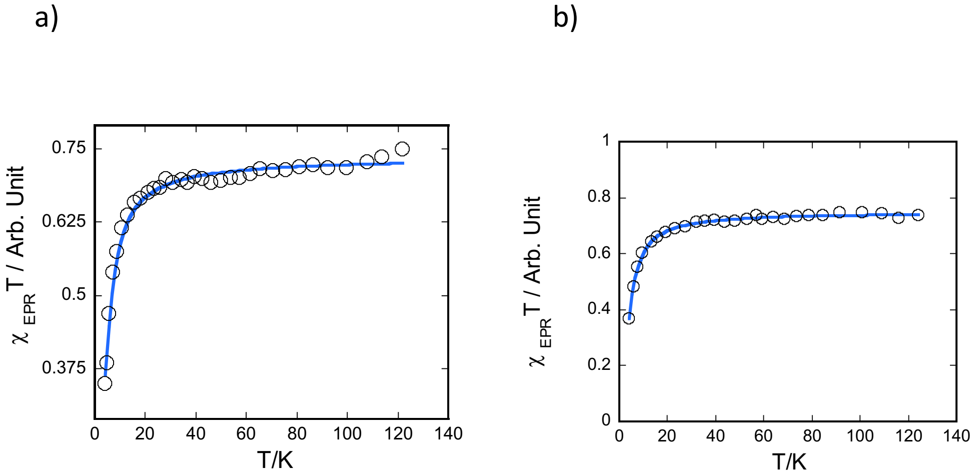

Figure S18: Temperature dependence of the EPR susceptibility (χT product) in CH_2Cl_2 frozen solution for a) **5** and b) **4.**

DFT calculations

This section reports the DFT-computed ^{14}N hyperfine and nuclear quadrupole principal values for **1**, **2** and **3** using the B3LYP functional with the CP(PPP) basis for the metal centre and the EPR-II basis set for others centers. The Singly Localized Molecular Orbitals (SOMOs) and the optimized structures are also provided and were calculated using the B3LYP functional with the 6-31g* basis set for all atoms.

Compound 1

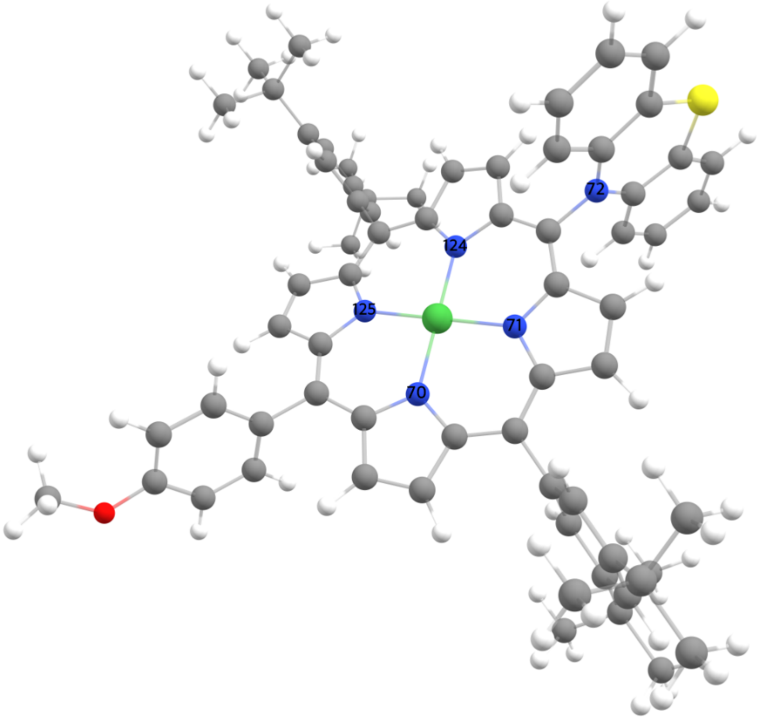

Atom labelling for mono-oxidized compound **1**

Hyperfine coupling constant

Center	A_{min} (MHz)	A_{mid} (MHz)	A_{max} (MHz)	A_{iso} (MHz)
N70	-0.01	0.01	0.03	0.01
N71	0.23	0.28	0.54	0.35
N72	0.97	1.12	42.86	14.98
N124	0.24	0.28	0.55	0.35
N125	-0.01	0.02	0.03	0.01

Quadrupole coupling constants

Center	V_{min} (a.u)	V_{mid} (a.u.)	V_{max} (a.u.)	e^2qQ (MHz)	eta
N70	-0.20	-0.24	0.44	1.99	0.09
N71	-0.21	-0.26	0.47	2.14	0.10
N72	0.31	0.39	-0.71	-3.21	0.12
N124	-0.21	-0.26	0.47	2.14	0.12
N125	-0.20	-0.24	0.44	1.99	0.09

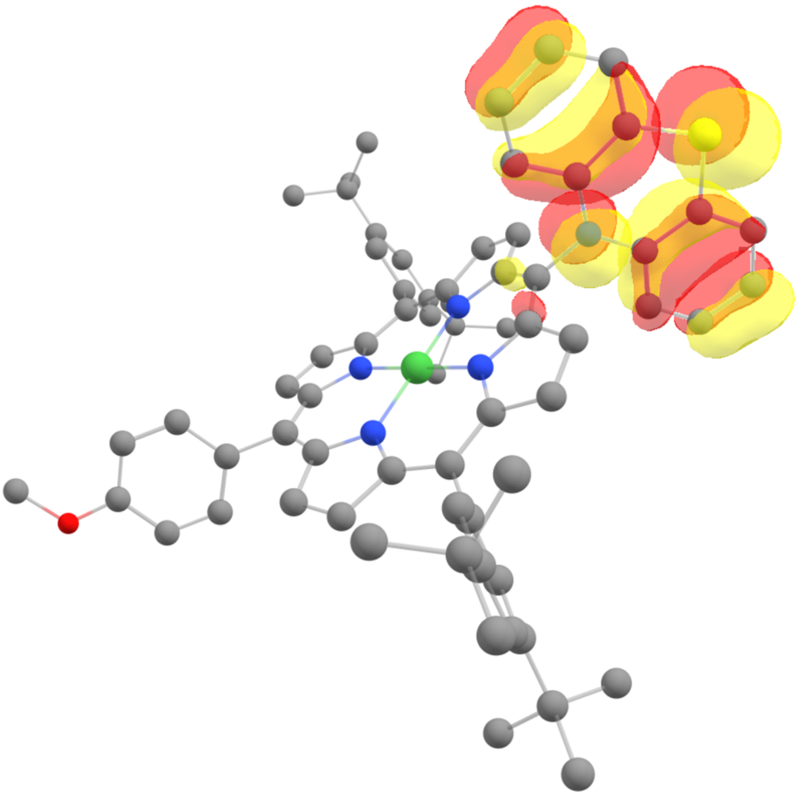

Localized SOMO for **1**.

Cartesian coordinates (Å) of the DFT-optimized structure of mono-oxidized compound **1**

C	1.701092	22.712718	-2.512058
C	2.502092	21.536611	-2.720993
H	3.008805	21.288002	-3.638232
C	2.498813	20.844828	-1.557116
H	2.995572	19.915432	-1.333892
C	1.651855	21.563109	-0.644677
C	1.299656	21.073187	0.611877
C	0.285219	21.646913	1.363313
C	-0.252793	21.063481	2.564059
H	0.125940	20.175457	3.041370
C	-1.309513	21.822406	2.938045
H	-1.957892	21.676396	3.786632
C	-1.390486	22.903728	1.994500
C	-2.269118	23.969993	2.096327
C	-2.922109	24.428129	4.405638
C	-1.632849	24.963803	4.613026
H	-0.925063	24.976650	3.799278
C	-1.265365	25.469882	5.839571
H	-0.269289	25.873273	5.967035
C	-2.163817	25.467742	6.917407
H	-1.867629	25.867172	7.878444
C	-3.429709	24.953381	6.745054
H	-4.135765	24.945292	7.566341
C	-3.820388	24.433348	5.502091
C	-5.568398	23.264861	3.766365
C	-6.800031	22.703618	3.397757

H	-7.592716	22.640255	4.133060
C	-6.996968	22.238289	2.116487
H	-7.949020	21.806266	1.837496
C	-5.956001	22.328442	1.180026
H	-6.103079	21.963895	0.171733
C	-4.741027	22.876565	1.525373
H	-3.955848	22.934780	0.788271
C	-4.509206	23.363457	2.829791
C	1.986241	19.845937	1.129588
C	3.232202	19.957958	1.743644
H	3.680636	20.939889	1.830674
C	3.894617	18.833688	2.244671
C	3.267795	17.591168	2.103806
H	3.765751	16.713587	2.483822
C	2.020541	17.441498	1.487526
C	1.390862	18.591850	1.004349
H	0.425577	18.522792	0.517984
C	5.266787	18.996737	2.923693
C	6.267157	19.608711	1.918108
H	7.246862	19.730869	2.388181
H	5.940713	20.589508	1.568039
H	6.387905	18.961374	1.045769
C	5.842604	17.658406	3.417644
H	5.194694	17.183155	4.157781
H	6.810703	17.835000	3.891620
H	5.999609	16.954742	2.596997
C	5.128770	19.938486	4.141249
H	4.426118	19.529597	4.871803
H	4.775023	20.928995	3.849366
H	6.097349	20.060740	4.633457
C	1.338058	16.071828	1.321144
C	2.159269	14.926223	1.936525
H	3.139049	14.825678	1.463912
H	1.628212	13.982530	1.793795
H	2.308269	15.062762	3.010191
C	-0.043700	16.094728	2.010424
H	0.057138	16.296395	3.079980
H	-0.537896	15.126526	1.892979
H	-0.698399	16.856371	1.583480
C	1.151218	15.776127	-0.183951
H	0.530649	16.530942	-0.670491
H	0.667162	14.805230	-0.320087
H	2.115242	15.749249	-0.698184
N	1.169120	22.707800	-1.238690
N	-0.419683	22.787421	1.030673
N	-3.269751	23.918314	3.153400
S	-5.448356	23.815296	5.416205
Ni	-0.081678	24.007537	-0.473596
C	-2.219083	25.116822	1.322020
C	-3.001998	26.298543	1.561345
H	-3.772435	26.404469	2.307712
C	-2.558330	27.235971	0.690997
H	-2.899135	28.251856	0.581208
C	-1.536128	26.620129	-0.112469
C	-0.917740	27.245606	-1.184644
C	-0.101892	26.548275	-2.072100

C	0.394456	27.109922	-3.298525
H	0.263513	28.134433	-3.604424
C	1.016749	26.109976	-3.966478
H	1.507163	26.155654	-4.924328
C	0.940946	24.938639	-3.135564
C	1.575742	23.738290	-3.444211
C	-1.203972	28.695127	-1.437957
C	-2.251360	29.071377	-2.276661
H	-2.844748	28.294969	-2.744039
C	-2.540843	30.418131	-2.511368
C	-1.743054	31.376049	-1.876296
H	-1.953844	32.419804	-2.046139
C	-0.683904	31.033783	-1.029647
C	-0.428339	29.675144	-0.822334
H	0.382837	29.364132	-0.175639
C	-3.715587	30.797939	-3.430599
C	-5.032574	30.274216	-2.813890
H	-5.879773	30.537904	-3.452902
H	-5.022028	29.188298	-2.703064
H	-5.202533	30.712518	-1.827210
C	-3.846680	32.318107	-3.621206
H	-2.951322	32.750575	-4.073989
H	-4.687886	32.527744	-4.285616
H	-4.038047	32.833715	-2.677080
C	-3.514613	30.156858	-4.821630
H	-2.587858	30.508125	-5.281617
H	-3.471748	29.067613	-4.765553
H	-4.344315	30.423593	-5.481979
C	0.196537	32.090193	-0.337562
C	-0.262561	33.527702	-0.637070
H	-1.282242	33.711271	-0.289882
H	0.393733	34.231526	-0.120485
H	-0.216517	33.757620	-1.703934
C	1.654628	31.938289	-0.826204
H	1.721599	32.089178	-1.906825
H	2.294194	32.679900	-0.339973
H	2.055394	30.949164	-0.598310
C	0.148546	31.882918	1.193042
H	0.523174	30.899692	1.482581
H	0.764582	32.635269	1.693266
H	-0.873902	31.979270	1.566619
N	-1.337137	25.313594	0.288460
N	0.253895	25.220476	-1.974315
C	2.200773	23.584323	-4.792938
C	3.589693	23.656720	-4.965602
C	1.408187	23.374244	-5.920299
H	4.229850	23.830157	-4.108726
H	0.331142	23.314341	-5.814548
C	4.161700	23.521730	-6.219879
C	1.968842	23.229570	-7.188516
H	5.235269	23.585377	-6.349748
H	1.319754	23.060396	-8.035996
C	3.354306	23.303584	-7.342175
C	3.235091	22.949354	-9.716099
H	3.954780	22.880950	-10.528116
H	2.674059	22.013946	-9.645887

H	2.548051	23.777712	-9.906879
O	4.006201	23.179332	-8.532932

Compound 2

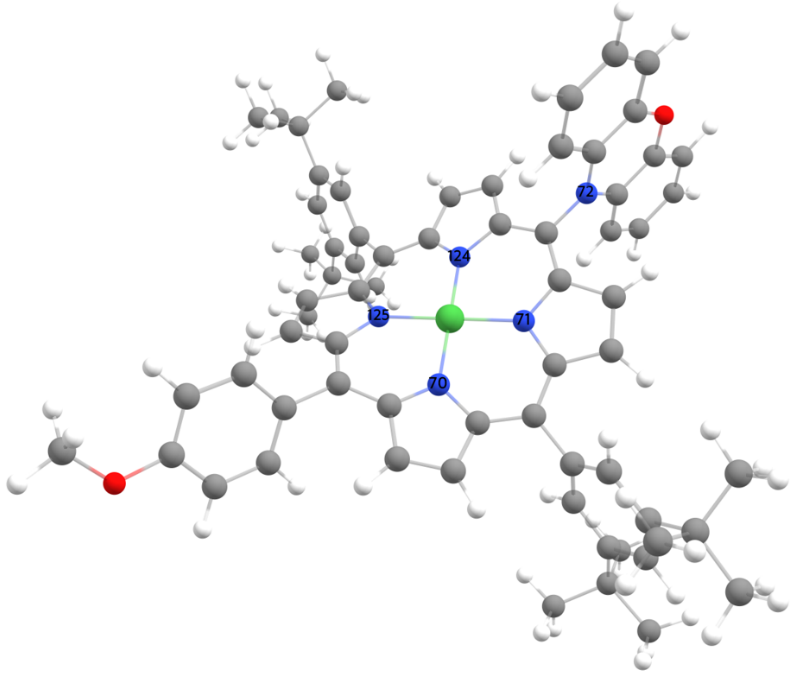

Atom labelling for mono-oxidized compound **2**

Hyperfine coupling constant

Center	A_{min} (MHz)	A_{mid} (MHz)	A_{max} (MHz)	A_{iso} (MHz)
N70	-0.01	0.02	0.04	0.02
N71	0.31	0.37	0.69	0.46
N72	1.04	1.24	51.18	17.82
N124	0.34	0.40	0.74	0.50
N125	0.01	-0.01	0.04	0.01

Quadrupole coupling constants

Center	V_{min} (a.u)	V_{mid} (a.u.)	V_{max} (a.u.)	e^2qQ (MHz)	eta
N70	-0.20	-0.25	0.45	2.04	0.10
N71	-0.22	-0.27	0.48	2.20	0.11
N72	0.29	0.34	-0.63	-2.85	0.08
N124	-0.21	-0.27	0.48	2.20	0.12
N125	-0.21	-0.24	0.45	2.02	0.07

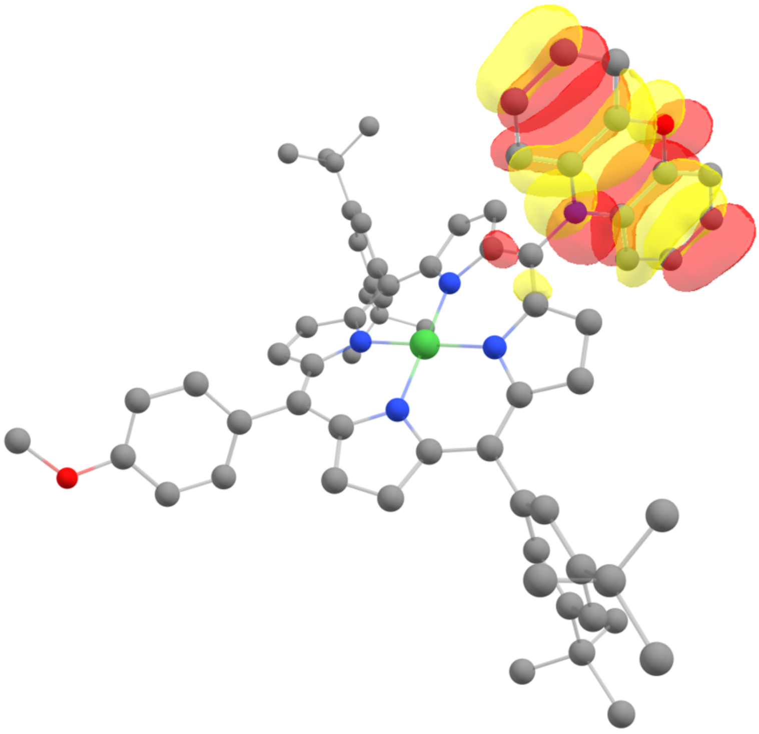

Localized SOMO for **2**.

Cartesian coordinates (Å) of the DFT-optimized structure of mono-oxidized compound **2**

C	1.540249	22.614950	-2.579296
C	2.387450	21.463227	-2.747758
H	2.853708	21.163347	-3.674060
C	2.497116	20.872175	-1.527673
H	3.055807	19.987193	-1.261020
C	1.641290	21.603863	-0.632388
C	1.305107	21.142441	0.646107
C	0.173118	21.623256	1.297329
C	-0.442732	20.985479	2.433558
H	-0.028219	20.145847	2.970978
C	-1.631298	21.610263	2.646345
H	-2.370521	21.389014	3.403273
C	-1.698473	22.687726	1.693913
C	-2.647547	23.704824	1.712480
C	-3.712886	24.000431	3.918496
C	-2.555087	24.598490	4.457474
H	-1.677697	24.721331	3.835416
C	-2.551299	25.022182	5.774464
H	-1.657327	25.481499	6.181909
C	-3.691276	24.863405	6.588676
H	-3.671791	25.201775	7.618835
C	-4.840298	24.275937	6.080161
H	-5.732395	24.138586	6.680770
C	-4.849293	23.847136	4.753329
C	-6.056010	22.834716	3.004217

C	-7.251648	22.252430	2.584517
H	-8.071151	22.172750	3.289825
C	-7.353269	21.794150	1.279308
H	-8.277565	21.338911	0.940492
C	-6.263222	21.918288	0.393416
H	-6.353633	21.557538	-0.625214
C	-5.074967	22.495427	0.805127
H	-4.241117	22.588901	0.121135
C	-4.948497	22.966550	2.127928
C	2.108474	20.035758	1.254840
C	3.370704	20.318202	1.790018
H	3.733361	21.340777	1.750660
C	4.152442	19.312925	2.376816
C	3.632419	18.009751	2.397366
H	4.223777	17.222417	2.843762
C	2.377084	17.686107	1.858533
C	1.623444	18.723020	1.290332
H	0.649817	18.516809	0.855667
C	5.530129	19.667359	2.970304
C	6.435809	20.244478	1.855559
H	7.415756	20.515710	2.266514
H	6.002671	21.142987	1.404569
H	6.592022	19.508063	1.059029
C	6.242603	18.445427	3.582583
H	5.664592	18.001786	4.401038
H	7.210541	18.755533	3.990985
H	6.432149	17.667294	2.834864
C	5.352177	20.728333	4.083744
H	4.717074	20.345061	4.891003
H	4.895919	21.647723	3.701997
H	6.326073	20.991807	4.512679
C	1.816079	16.250852	1.864583
C	2.768258	15.246761	2.542373
H	3.733174	15.186793	2.026642
H	2.319890	14.247481	2.520094
H	2.954240	15.503880	3.591251
C	0.469708	16.225882	2.626938
H	0.604246	16.540439	3.668312
H	0.055656	15.210625	2.629644
H	-0.272101	16.886672	2.166373
C	1.586216	15.786539	0.405719
H	0.879939	16.436029	-0.122011
H	1.179635	14.768345	0.392979
H	2.526992	15.784225	-0.156872
N	1.062966	22.675504	-1.280907
N	-0.604561	22.683201	0.859174
N	-3.781818	23.555902	2.602794
O	-5.992095	23.275270	4.288878
Ni	-0.218604	23.912457	-0.570570
C	-2.511251	24.910846	1.032759
C	-3.278129	26.098336	1.302604
H	-4.129360	26.164688	1.965180
C	-2.681029	27.104194	0.609199
H	-2.956602	28.147848	0.584746
C	-1.607112	26.516645	-0.149659
C	-0.871728	27.189445	-1.121596

C	-0.087897	26.480759	-2.039361
C	0.398421	27.047946	-3.268706
H	0.330956	28.091924	-3.536464
C	0.894481	26.019384	-4.007315
H	1.340028	26.058168	-4.989979
C	0.769751	24.830489	-3.205536
C	1.347145	23.602786	-3.549239
C	-1.003624	28.671906	-1.278186
C	-2.172921	29.242927	-1.794523
H	-2.989018	28.586230	-2.080430
C	-2.291882	30.629922	-1.961057
C	-1.198930	31.428539	-1.590762
H	-1.274908	32.500190	-1.713198
C	-0.009678	30.892007	-1.072870
C	0.068943	29.499926	-0.926480
H	0.969948	29.041916	-0.529891
C	-3.591541	31.217934	-2.544972
C	-4.784939	30.830102	-1.639177
H	-5.718236	31.232181	-2.050627
H	-4.898398	29.743798	-1.556587
H	-4.656214	31.234095	-0.628469
C	-3.550071	32.754396	-2.650433
H	-2.743133	33.096769	-3.308018
H	-4.495030	33.117130	-3.069204
H	-3.418194	33.227604	-1.670768
C	-3.821663	30.644178	-3.964187
H	-2.996001	30.912489	-4.633319
H	-3.905358	29.552324	-3.953671
H	-4.748922	31.047251	-4.387619
C	1.189757	31.769725	-0.664839
C	0.936821	33.269503	-0.912532
H	0.084735	33.641945	-0.333008
H	1.819916	33.842057	-0.608235
H	0.751085	33.483256	-1.971011
C	2.436289	31.351537	-1.482013
H	2.262570	31.486736	-2.555596
H	3.298260	31.966357	-1.196430
H	2.703298	30.303641	-1.311103
C	1.478548	31.572528	0.843128
H	1.711336	30.529175	1.080087
H	2.337045	32.183709	1.146468
H	0.615748	31.872833	1.448944
N	-1.503280	25.163178	0.130795
N	0.162221	25.123385	-2.000103
C	1.842912	23.411257	-4.942650
C	3.210395	23.253043	-5.239431
C	0.935056	23.410756	-6.011323
H	3.941989	23.277165	-4.437520
H	-0.125247	23.533491	-5.810480
C	3.645742	23.091100	-6.548740
C	1.357367	23.242739	-7.332302
H	4.701198	22.978295	-6.775723
H	0.621214	23.239326	-8.127179
C	2.721843	23.080826	-7.607262
C	2.354103	22.891823	-9.967351
H	2.982683	22.745252	-10.846043

H	1.638717	22.065548	-9.886418
H	1.810674	23.839219	-10.059976
O	3.246281	22.914211	-8.852596

Compound 3

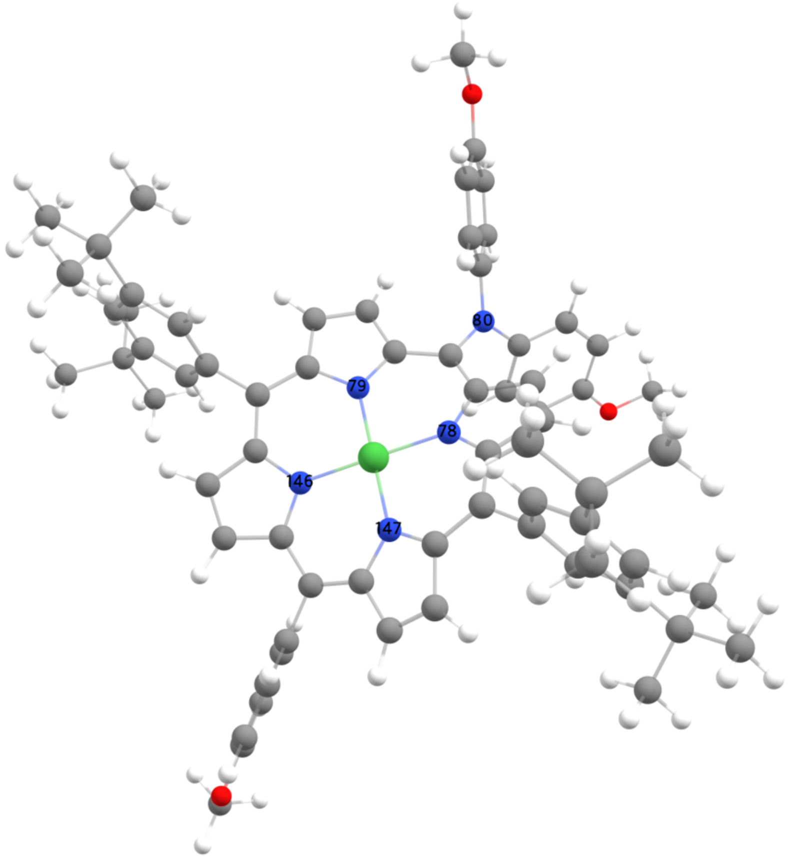

Atom labelling for mono-oxidized compound **3**

Hyperfine coupling constant

Center	A_{min} (MHz)	A_{mid} (MHz)	A_{max} (MHz)	A_{iso} (MHz)
N78	-0.23	-0.23	3.20	0.92
N79	0.18	0.28	3.97	1.48
N80	1.32	1.36	42.57	15.1
N146	0.11	0.17	3.26	1.18
N147	0.21	0.27	3.40	1.29

Quadrupole coupling constants

Center	V_{min} (a.u)	V_{mid} (a.u.)	V_{max} (a.u.)	e^2qQ (MHz)	eta
N78	-0.21	-0.24	0.46	2.07	0.05
N79	-0.21	-0.25	0.46	2.10	0.11
N80	0.40	0.41	-0.81	-3.68	0.02
N146	-0.20	-0.25	0.44	2.01	0.12
N147	-0.20	-0.25	0.45	2.02	0.12

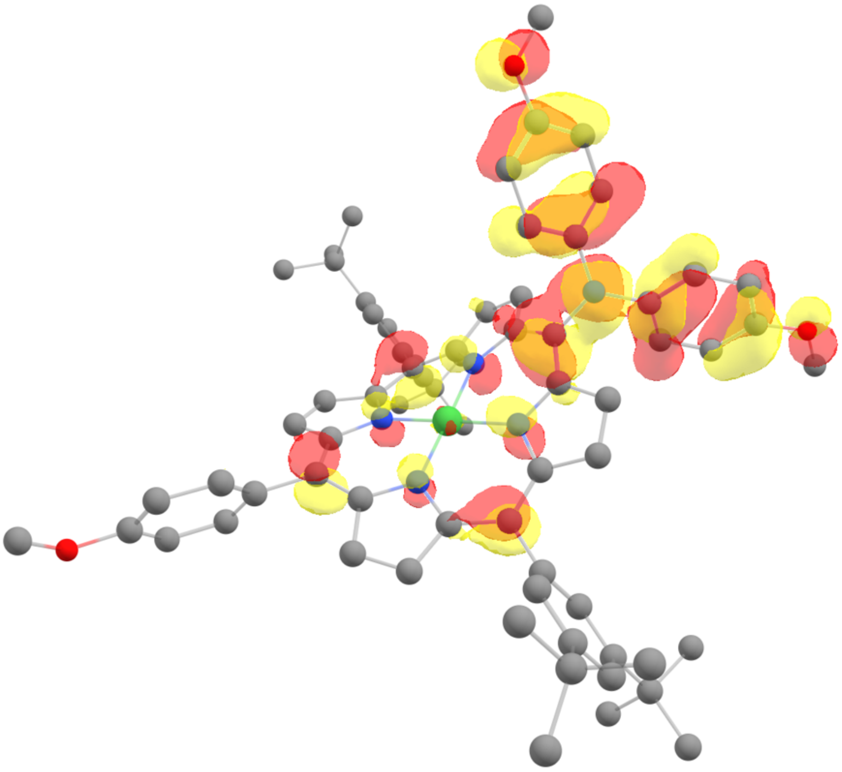

Localized SOMO for **3**.

Cartesian coordinates (Å) of the DFT-optimized structure of mono-oxidized compound **3**

C	3.624142	3.701951	11.507005
C	2.995276	3.462393	12.778596
H	2.682418	2.494465	13.141470
C	2.932971	4.659940	13.421358
H	2.533599	4.864619	14.403885
C	3.456258	5.642118	12.507538
C	3.318699	7.029851	12.690032
C	3.391612	7.934296	11.617828
C	2.973709	9.310980	11.647843
H	2.625013	9.848831	12.515676
C	3.073847	9.772903	10.373095
H	2.839501	10.760536	10.004992

C	3.633691	8.709678	9.581895
C	4.104791	8.886864	8.283698
C	3.915609	7.250757	15.032369
C	5.302835	7.264922	14.759825
H	5.654113	7.493036	13.759600
C	6.213236	7.027408	15.770006
H	7.279925	7.054522	15.574779
C	5.771388	6.770993	17.085426
C	4.391317	6.748654	17.362769
H	4.025427	6.533255	18.358864
C	3.477640	6.983427	16.344831
H	2.416748	6.935220	16.562922
C	6.369568	6.325936	19.363655
H	5.811922	7.176639	19.768240
H	7.308402	6.209323	19.903590
H	5.774660	5.411382	19.453379
C	1.767060	8.144333	14.240643
C	1.683748	9.212117	15.162740
H	2.576666	9.564957	15.666432
C	0.471288	9.829493	15.400581
H	0.396942	10.658229	16.096626
C	-0.693768	9.401732	14.728636
C	-0.613266	8.346967	13.800660
H	-1.493536	7.994447	13.277892
C	0.606963	7.729006	13.561780
H	0.658931	6.900855	12.863307
C	-3.053356	9.676819	14.408619
H	-2.991941	9.810428	13.323821
H	-3.813527	10.339938	14.819824
H	-3.302132	8.636903	14.643789
C	3.733278	10.137069	7.549448
C	2.448692	10.268072	7.018800
H	1.751990	9.442854	7.130987
C	2.057022	11.436342	6.341214
C	2.995175	12.467877	6.235526
H	2.713657	13.381389	5.726195
C	4.297104	12.375403	6.764661
C	4.652489	11.191694	7.417024
H	5.639545	11.073040	7.848318
C	0.640250	11.530249	5.742602
C	-0.411215	11.330307	6.860579
H	-0.307028	10.358123	7.353558
H	-1.422111	11.384782	6.439593
H	-0.319413	12.109121	7.626369
C	0.470168	10.423995	4.672321
H	1.194950	10.552412	3.860206
H	-0.536593	10.466475	4.239794
H	0.611743	9.423907	5.095163
C	0.371253	12.892360	5.074229
H	0.454527	13.719842	5.788057
H	-0.646383	12.904811	4.669051
H	1.060518	13.082595	4.244222
C	5.263594	13.566769	6.618312
C	4.662745	14.804296	7.329678
H	5.337488	15.662544	7.227747
H	4.518523	14.609916	8.398630
H	3.694493	15.087176	6.904013
C	5.465932	13.889004	5.117625
H	4.524956	14.156865	4.626179

H	5.892409	13.030438	4.586360
H	6.154003	14.735015	5.003813
C	6.645060	13.281049	7.237563
H	7.135475	12.423458	6.763271
H	6.577100	13.085462	8.313558
H	7.294343	14.152373	7.098806
N	3.885251	5.054877	11.347838
N	3.814763	7.576599	10.358304
N	2.995989	7.496266	13.991872
O	6.739542	6.562396	17.998733
O	-1.826168	10.062781	15.041315
Ni	4.621247	5.917216	9.806078
C	5.008550	7.979191	7.714138
C	5.765602	8.239737	6.521405
H	5.627728	9.092962	5.874085
C	6.675548	7.233041	6.406611
H	7.414946	7.088697	5.632910
C	6.426918	6.320303	7.490050
C	7.017065	5.051633	7.579443
C	6.441889	4.053202	8.375139
C	6.772280	2.655555	8.294529
H	7.588201	2.237246	7.725596
C	5.845014	1.987478	9.034545
H	5.759699	0.922810	9.193153
C	5.012457	2.982545	9.650416
C	4.087749	2.697294	10.665652
C	8.165972	3.773883	5.713903
H	7.244162	3.235348	5.517402
C	9.285631	3.504844	4.938933
H	9.252323	2.758981	4.150903
C	10.485090	4.202670	5.160123
C	10.536724	5.186262	6.157276
H	11.446532	5.742336	6.348950
C	9.399516	5.454253	6.922195
H	9.454777	6.213159	7.697296
C	12.779335	4.518662	4.547623
H	13.168325	4.347422	5.557737
H	13.458535	4.081111	3.815687
H	12.689185	5.595837	4.366870
C	3.696505	1.277781	10.938270
C	4.533898	0.432040	11.667525
H	5.480367	0.821180	12.030815
C	4.164797	-0.896808	11.939334
C	2.932792	-1.340565	11.447318
H	2.628829	-2.361761	11.643003
C	2.064142	-0.516945	10.705935
C	2.464473	0.800629	10.463596
H	1.832086	1.476678	9.899778
C	5.114386	-1.798199	12.751120
C	6.444137	-1.964454	11.975656
H	6.931976	-1.001065	11.794486
H	7.140311	-2.590026	12.547005
H	6.272878	-2.444811	11.005531
C	5.400287	-1.142488	14.123866
H	4.473845	-1.003230	14.692602
H	6.069002	-1.780219	14.713949
H	5.880930	-0.164553	14.018081
C	4.526446	-3.200211	13.002294
H	4.339063	-3.739228	12.067055

H	5.237419	-3.793274	13.587979
H	3.587946	-3.154539	13.566326
C	0.724777	-1.084948	10.197676
C	0.994691	-2.290664	9.264897
H	0.045999	-2.701389	8.899296
H	1.591430	-1.988607	8.396830
H	1.530078	-3.094784	9.780203
C	-0.126060	-1.552857	11.402941
H	0.386249	-2.322938	11.988684
H	-0.352434	-0.714218	12.071601
H	-1.075378	-1.975769	11.053389
C	-0.092851	-0.045269	9.407098
H	-0.351170	0.824997	10.020888
H	0.445532	0.306906	8.520072
H	-1.030061	-0.499851	9.068244
N	5.403439	6.788570	8.289478
N	5.375228	4.245649	9.234507
C	8.202018	4.749728	6.729663
O	11.528058	3.856854	4.358234
